# Solid-Liquid Interface Lubricating Hydrogels for Tendon-Bone Healing

**DOI:** 10.34133/research.0924

**Published:** 2025-10-23

**Authors:** Pengzhen Zhuang, Liang Chen, Yu Zhang, Wu Yang, Yu Chen, Longxi Wu, Lei Xiang, Zhen Wang, Jessica M. Rosenholm, Guilai Zuo, Tingjun Ye, Hongbo Zhang, Wenguo Cui

**Affiliations:** ^1^ Department of Orthopaedics, Shanghai Key Laboratory for Prevention and Treatment of Bone and Joint Diseases, Shanghai Institute of Traumatology and Orthopaedics, Ruijin Hospital, Shanghai Jiao Tong University School of Medicine, Shanghai 200025, P.R. China.; ^2^ Pharmaceutical Sciences Laboratory, Faculty of Science and Engineering, Åbo Akademi University, Turku 20520, Finland.; ^3^ School of Nursing, Shanghai Jiao Tong University, Shanghai 200025, P.R. China.; ^4^ Department of Orthopedic Oncology, The Affiliated Hospital of Qingdao University, Qingdao, Shandong 266000, P.R. China.

## Abstract

A solid-liquid interface lubrication system composed of tissue and synovial fluid ensures the physiological functions of the tendon-bone (TB). However, after the TB tears, stress concentration and friction disrupt the solid-liquid interface lubrication of the TB and disturb its healing. Herein, solid-liquid interface lubrication hydrogels (MnCaP/HS) are constructed by encapsulating ion-coordinated calcium manganese phosphate microgels into covalent/ionic-cross-linked hyaluronic acid/sodium alginate dual networks. Solid lubrication interfaces are thus formed at the damaged TB through surface hydrogen bonding and ionic toughening and continuously release the biolubrication components into tissue fluid for rolling lubrication layers and liquid lubrication films. This long-lasting lubricating property and MnCaP synergistically improve the immune-regenerative microenvironment of the TB. In vitro, MnCaP/HS increases the toughness by 600% and reduces the friction coefficient at the solid-liquid interface by 60% compared with those of the phosphate-buffered saline group. Moreover, it releases metal ions to promote the anti-inflammation of macrophages and the cartilage-bone gradient differentiation of bone marrow mesenchymal stem cells. In vivo, the maximum load and stiffness of tendons in the MnCaP/HS group increase by 102% and 75%, respectively while exerting long-lasting anti-inflammation to inhibit scarring and complete microstructural reconstruction. Thus, MnCaP/HS provides new solutions for TB reconstruction by providing long-lasting lubrication and a regenerative microenvironment.

## Introduction

More than 30 million people worldwide suffer from shoulder disorders annually, with tendon tears (tendon-bone [TB]) being the most common type [1]. TB interfaces have continuous transitional structures composed of tendon, cartilage, and bone, and this soft-to-hard gradient effectively relieves stress concentrations during movement [2]. However, forceful movement and sustained stress concentrations could lead to TB interface tears. Once tendons are ruptured, muscular contraction forces cannot effectively transmit to the bone, which limits shoulder function and is accompanied by severe pain and inflammatory response. In clinical practice, the bone is usually immobilized using a medical immobilizer, and then surgical sutures are used to reanchor the tendon to the bone in conjunction with medication for healing; however, they exhibit failure rates ranging from 20% to 94%, depending on the hospital and the surgeon [3]. It is widely accepted that the major limiting factor in TB healing is the inability of the nascent fibrous scar tissue to provide adequate tensile strength, resulting in failure to distribute stress and high friction between the injured tendon and adjacent structures [4,5]. Specifically, the subacromial bursa is usually excised during TB reconstruction surgery, which results in the loss of its mechanical cushioning effect between the acromion and the tendon, thereby exacerbating stress concentrations and high frictional wear between the tissues [6]. Such excessive friction further triggers a persistent inflammatory response and disrupts the regenerative microenvironment, further delaying the healing process at the TB interface [7]. Therefore, reducing long-term high frictional wear at the TB interface is essential for promoting effective healing.

Biolubrication can reduce excessive friction between tissues, and its core principle involves maintaining an efficiently lubricated sliding interface with an ultralow coefficient of friction (COF), thus preventing shear-induced tissue damage [8]. The outer surface of the tendon within the synovium is the tendon membrane, whose exposed boundary layer contains hyaluronic acid, phospholipids, proteoglycans, and lubricating protein solutions that confer high lubricity [9,10]. However, following tendon rupture, the synovial tissues are infiltrated with many inflammatory cells, potentially leading to fibrosis and adhesions within the synovial membrane, which restrict the free sliding motion of the tendon. Additionally, subacromial impingement exacerbates the inflammatory response and the risk of tendon rupture [11]. Therefore, it is critical to effectively prevent tendon damage caused by acromial impingement and optimize the tendon’s ability to slide freely at the interface. To address this challenge, several lubrication strategies have been used in the field of tendon therapy, including liquid lubrication and solid lubrication. Although liquid lubricants (phosphate-buffered saline [PBS], hyaluronic acid solution, bovine slippery fluid, and 5-fluorouracil) have been shown to be effective, their rapid metabolism results in the need for multiple injections, which not only increases the risk of treatment failure but also decreases patient compliance [12–15]. In contrast, single injections of lubricating gels (carboxymethylcellulose and polyethylene oxide) have shown potential to reduce tissue adhesions and restore tendon function through hydrating lubricating layers [16]. Polyethylene glycol polyester and hyaluronic acid hydrogels also reduce tendon friction with surrounding tissues by virtue of their abundance of polar groups that build hydrogen bonds with water molecules and form lubricating layers [17,18]. Furthermore, lubricin, hyaluronic acid, phospholipids, and their compounds have been chemically modified to form hydrated lubricating coatings on biomaterial surfaces, enhancing their frictional properties and suitability for biomedical applications [19–22]. Despite these advancements in hydrogels and lubricant coating applications for tendon healing, under high contact pressure, their metabolism is too fast, and the low friction coefficient that depends on the hydration layer is difficult to maintain for a long time. Furthermore, significant challenges remain in absorbing frictional energy and maintaining long-term lubrication performance at the interface during movement. Therefore, we propose to develop a solid-liquid interface lubrication strategy. The solid lubrication performance of the hydrogels at the TB interface is enhanced by the multilevel energy dissipation of the hydrogels in the pre-healing phase, and continuous solid-liquid lubrication interfaces are formed to provide long-lasting lubrication during the healing process. This jointly reduces the stress damage at the TB interface and thus provides a favorable regenerative microenvironment.

Although the solid-liquid interface lubrication strategy alleviates stress-induced injury at the TB interface, reconstructing its microstructural integrity remains challenging. Thus, modulation of the cellular gradient at the TB interface, including immune responses and stem cell differentiation, is critical for accelerated healing. Inflammatory macrophages accumulate at the site of the injured TB interface and secrete excessive inflammatory cytokines, which compromise the osteogenic and chondrogenic differentiation capacity of bone marrow mesenchymal stem cells (BMSCs). Additionally, excessive formation of fibrous scar tissue further delays restoration of the cellular gradient at the interface [2,23]. Our previous studies demonstrated that metal ions and calcium phosphate dynamically modulate cellular behavior at tissue interfaces, guiding tissue regeneration [24–26], and metal ions (Sr, Mg, Mn, etc.) have been widely reported to promote osteogenesis and chondrogenesis [27,28]. Manganese is an essential trace element, and compared to Sr and Mg, it has been shown to promote the transition of macrophages from the M1 state to the M2 state in various manganese-based nanomaterials by mimicking antioxidant enzymes (superoxide dismutase [SOD] and catalase), thereby reducing the secretion of inflammatory cytokines [29,30]. This is particularly important at the TB interface due to high-intensity friction processes. Additionally, elemental Mn has been reported to enhance chondrogenic differentiation of BMSCs, making it particularly suitable for repairing soft-to-hard tissue interfaces [31].

Therefore, we hypothesize that introducing Mn-doped calcium phosphate (MnCaP) within a sustained lubrication strategy may synergistically establish ordered pro-inflammatory-to-anti-inflammatory gradients among macrophages and facilitate the bidirectional gradient differentiation of BMSCs, thereby remodeling the regenerative microenvironment and accelerating microstructural reconstruction at the TB interface. TB injury models were created and implanted with MnCaP/HS hydrogels, which provide continuous solid-liquid interface lubrication during the TB healing cycle. The solid lubrication interface formed by ionic bond toughening and hydrogen bonding could resist tissue stress injury and reduce early inflammatory response, and the rolling lubrication layer and liquid lubrication film formed by hydrogel degradation could synergistically improve the immune regeneration microenvironment with MnCaP, which promoted the formation of an anti-inflammatory macrophage gradient and the chondrocyte-bone gradient differentiation of BMSCs, and thus suppressed the long-term inflammation and the formation of scar tissue, and finally achieved efficient microstructural reconstruction of the TB interface. Ultimately, this achieves the synergistic effect of “solid-liquid lubrication to reduce mechanical stimulation and thereby reduce inflammation initiation, with MnCaP further inhibiting excessive inflammation and promoting cell gradient reconstruction”.

## Results and Discussion

### Fabrication and characterization of MnCaP

**Fig. 1.**
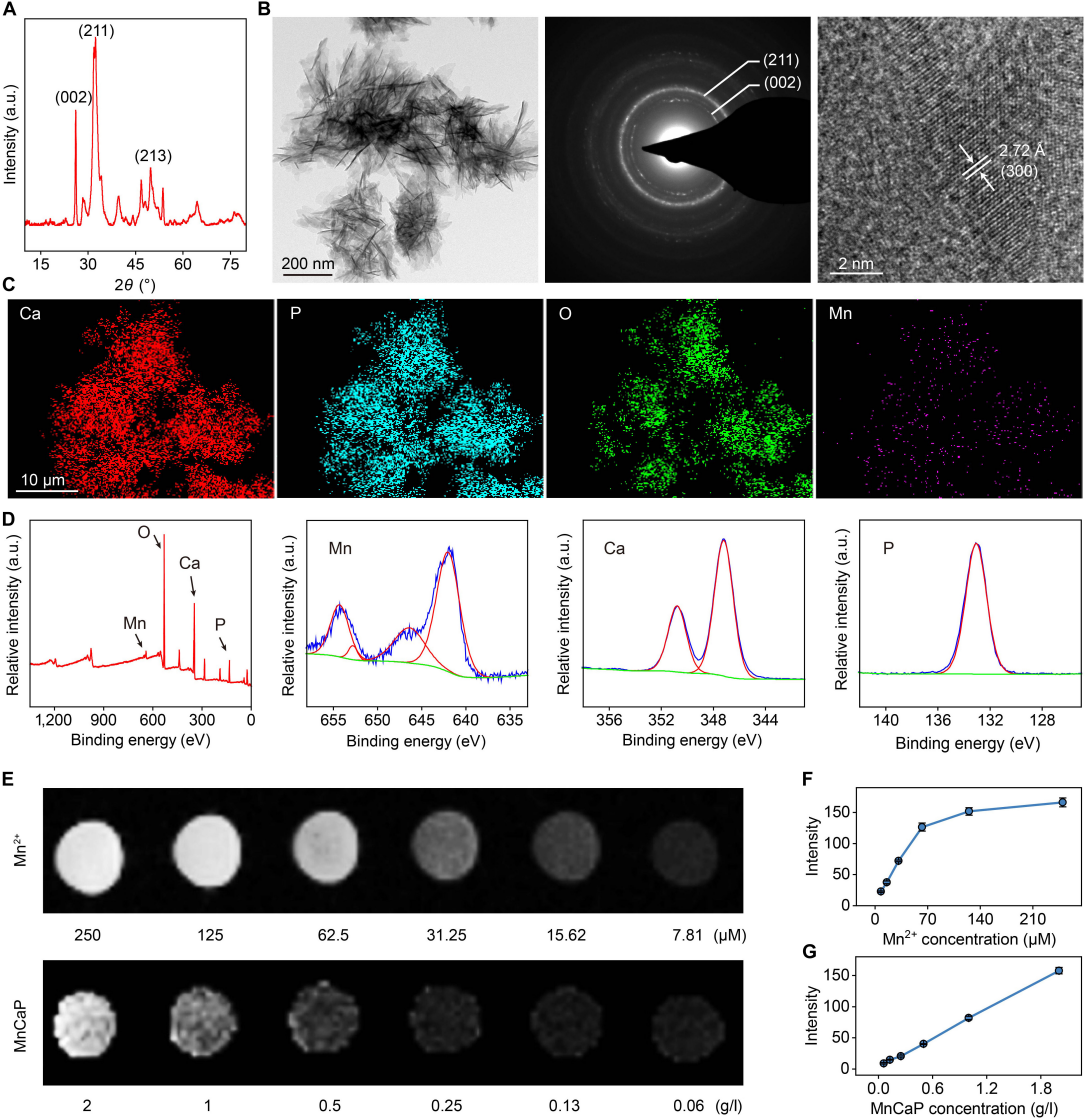
Physicochemical characterization of calcium manganese phosphate nanosheets. (A) X-ray diffraction (XRD) pattern, (B) transmission electron microscopy (TEM) image, (C) elemental distribution, and (D) x-ray photoelectron spectroscopy (XPS) pattern of calcium manganese phosphate nanosheets. (E) Nuclear magnetic resonance (NMR) imaging of calcium manganese phosphate and manganese ions and (F and G) their quantitative analysis.

Solid-liquid interface lubricating hydrogels, as TB repair materials, must have excellent energy dissipation, lubrication, and bioactivity properties, consisting of calcium manganese phosphate microgel and hyaluronic acid/sodium alginate (SA) dual-network hydrogels. The physical phase of MnCaP was confirmed to be attributed to hydroxyapatite using x-ray diffraction (XRD) with Powder Diffraction File card number 09-0432, and characteristic peaks of crystalline facets such as (211), (002), and (213) were detected, as shown in Fig. 1A. To understand the size and morphology of MnCaP nanosheets, we observed the synthesized samples using transmission electron microscopy (TEM) and scanning electron microscopy (SEM). TEM images showed the different orientations of the MnCaP nanosheets liner and confirmed that it was a stack of 2-dimensional nanosheets, and diffraction patterns further confirmed that it was polycrystalline, and the (211) and (002) crystal facets were consistent with the XRD results, and the high-resolution images demonstrated its (300) crystal facets with an interplanar spacing of 2.72 Å, which was consistent with hydroxyapatite crystal facet characteristics (Fig. 1B). The results of SEM combined with energy spectroscopy showed that the elements Ca, P, O, and Mn were uniformly distributed over the entire nanosheets (Fig. 1C and Fig. S1), and the characteristic peaks of the element Mn were also shown in the x-ray photoelectron spectroscopy (XPS) pattern, which indicated the successful doping of the element Mn (Fig. 1D). An important advantage of manganese-doped biomaterials lies in their ability to enhance magnetic resonance imaging (MRI) capabilities, which is suitable for the diagnosis and treatment of soft tissues such as tendons, so we utilized an MRI instrument to examine the in vitro imaging capabilities of Mn^2+^ and MnCaP, respectively (Fig. 1E). The results showed that Mn^2+^ greater than 60 μM and MnCaP nanosheets greater than 0.5 g/l had good MRI performance, and the quantitative data were consistent with the imaging results (Fig. 1F and G). Therefore, it is valuable to consider this threshold to design solid-liquid interface lubricating hydrogels for the diagnosis of tendon tissues.

### Fabrication and characterization of solid-liquid interface lubricating hydrogels

**Fig. 2.**
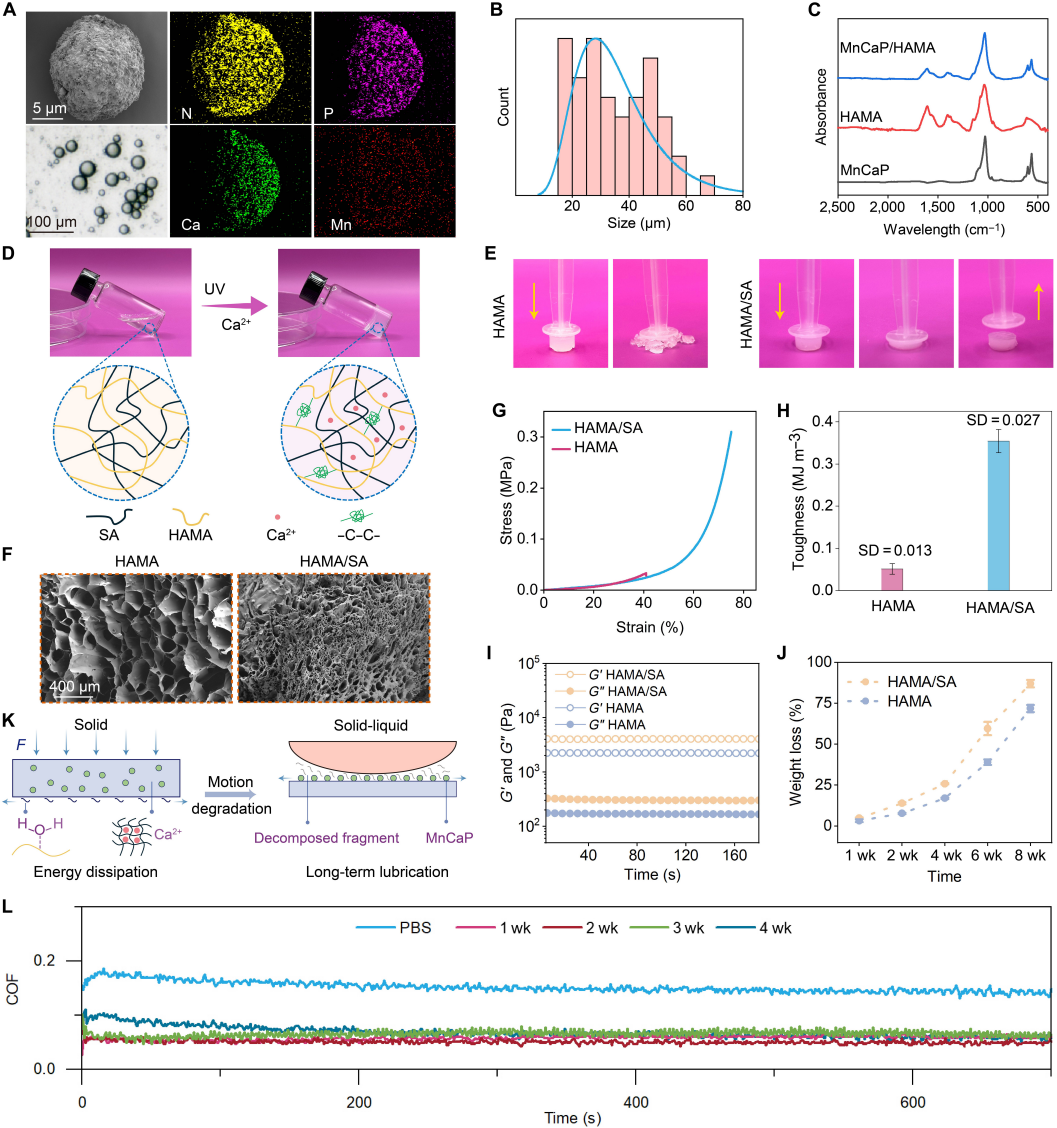
Physicochemical characterization of solid-liquid interface lubricating hydrogels. (A) Optical pictures, scanning electron microscopy (SEM) pictures, elemental distribution, (B) particle size distribution, and (C) infrared spectra of calcium manganese phosphate microgel. (D) Gel-forming mechanism and (E) compression properties of hyaluronic acid methacryloyl (HAMA)/sodium alginate (SA) composite hydrogel. (F) SEM pictures, (G) stress–strain curves, (H) toughness (*n* = 3), (I) *G*′/*G*″, and (J) weight loss of HAMA and HAMA/SA hydrogels. (K) Lubrication mechanism and (L) dynamic friction coefficient of solid-liquid interface lubricating hydrogels. UV, ultraviolet; COF, coefficient of friction; PBS, phosphate-buffered saline.

Further, we formed MnCaP microgels by compositing MnCaP nanosheets with hyaluronic acid-based hydrogels via emulsification. Hyaluronic acid is an important constituent of the extracellular matrix that improves lubricating properties through hydrogen bonding hydration. To enhance its maneuverability, we introduced methacrylic acid groups into the hyaluronic acid molecular chain to endow it with photoresponsiveness, and gel-forming experiments confirmed the photo-cross-linking properties of the hydrogels. We observed the surface morphology and size of the MnCaP microgel by SEM, which was denser and without a porous structure, and the microgel size was around 10 μm. In addition, the energy spectrum results indicated that the elements of N, P, Ca, and Mn were uniformly distributed on the surface of the microgel, indicating that the MnCaP microgel had a better composite process (Fig. 2A and B). The characteristic peaks of phosphate at 564 and 602 cm^−1^ were shown in the infrared curves, and these characteristic peaks also appeared for the composite microspheres, indicating that MnCaP had been successfully loaded into the microspheres (Fig. 2C).

Hyaluronic acid/SA dual-network hydrogels were prepared by ultraviolet light and calcium-ion cross-linking, with photo-cross-linked hyaluronic acid methacryloyl (HAMA) acting as the primary network to provide enough hydrogen bonds and calcium-ion cross-linking SA acting as the secondary network to increase the toughness (Fig. 2D). A downward force was applied to the HAMA hydrogel and HAMA/SA hydrogel, respectively, with 50% deformation as a benchmark. The molecular network of HAMA was more brittle and could not resist the deformation and ruptured, while the HAMA/SA hydrogel successfully resisted the deformation due to the toughening effect of ionic bonding (Fig. 2E). The SEM results indicated that the porosity inside the HAMA hydrogel was around 100 to 300 μm, whereas that of the HAMA/SA hydrogel was smaller and the wall thickness of the hydrogel was increased. This resulted in an overall denser behavior, which was associated with an increased polymer concentration as well as formed an ionic bonding-based double network (Fig. 2F) [32]. The stress–strain results indicated that the HAMA hydrogel fractured at 40.9% strain and the HAMA/SA hydrogel at 75.1%, with compressive strengths of 0.03 and 0.31 MPa, respectively. This demonstrated that the compressive properties of the hydrogels were considerably improved by introducing calcium-ion cross-linking of the SA network (Fig. 2G). The stress–strain curves corresponding to the integral area of toughness were 0.05 and 0.35 MJ m^−3^ for the HAMA hydrogel and HAMA/SA hydrogel, respectively, which indicated that the HAMA/SA hydrogel had significantly enhanced energy dissipation ability (Fig. 2H). The energy storage modulus (*G*′) and dissipation modulus (*G*″) of the HAMA/SA hydrogel were also higher than those of the HAMA hydrogel (Fig. 2I), which together imply that the HAMA/SA hydrogel could absorb more external energy and provide a cushioning region to counteract frictional wear, meeting the mechanical demands of TB interfaces for repair materials. Ionic cross-linking sites were shown to serve as “sacrificial bonds” to dissipate energy and increase hydrogel toughness [33]. Compared to the HAMA hydrogel, the HAMA/SA hydrogel had a faster degradation rate (Fig. 2J), which might be related to the degradation of the SA/calcium network and implied that it could rapidly and stably release bioactives to act at the TB interface. HAMA/SA hydrogel will be abbreviated as HS in the subsequent text.

Long-term high frictional wear in TB repairs is one of the most important reasons for their healing failure [6]; however, most of the current repair materials have not been tested for their long-term lubrication properties. It is difficult for those materials to match the lubrication needs during the healing cycle and increases the risk of TB interface fracture. To assess the long-term lubrication properties of MnCaP/HS, we conducted a 4-week degradation experiment in vitro and collected degradation products to assess their friction coefficients. The gradually turbid solution indicated that the hydrogel degradation products and released calcium manganese phosphate increased with time, and the concentrations of Mn and Ca ions at 4 weeks were 13.86 and 48.97 μg/ml, respectively (Fig. S2). Friction and wear results indicated that the COF of the PBS solution in the liquid state was 0.152, and the COFs of the solid-liquid interface lubricating hydrogel after 1, 2, 3, and 4 weeks of degradation were 0.068, 0.058, 0.051, and 0.066, respectively, which were obviously inferior to those of the PBS solution, possibly due to the formation of a rolling lubrication layer and a liquid lubrication film. In addition, we prepared HAMA solutions ranging from 10 to 80 μg/ml and measured their COFs, confirming that they can act as liquid lubricating films to reduce the COF (Fig. 2L and Fig. S3). The hyaluronic acid composition and spherical structure of the microgel provided support for rolling lubrication, as reported by Lei et al [34], while the hyaluronic acid molecules in the degradation solution formed a liquid lubrication film, which provided a hydrated lubrication layer to reduce the COF [35]. Therefore, the produced continuous solid-liquid-based lubrication interface could provide a long-lasting lubrication microenvironment for 28 d to meet the needs of the TB healing cycle (Fig. 2K).

### Biocompatibility assessment of solid-liquid interface lubricating hydrogels

BMSCs, chondrocytes, and macrophages are key cells involved in TB healing, and their osteogenic/chondrogenic and immunomodulatory functions determine the quality of TB healing [36]. Here, we investigated the biocompatibility of the solid-liquid interface lubricating hydrogels by conducting calcein AM/propidium iodide staining of BMSCs, chondrocytes, and Raw264.7 cells treated with the blank control (no materials involved), HS, CaP/HS, and MnCaP/HS groups, respectively (Fig. S4a). The results confirmed that there was a marked trend of increasing cell viability and almost no dead cells were detected in all groups. In addition, the morphology of BMSCs was further evaluated by actin/4′,6-diamidino-2-phenylindole (DAPI) staining, which showed that the cells in all groups were spindle shaped with extended pseudopods. Further, the proliferation of BMSCs, chondrocytes, and Raw264.7 cells in different groups after treatment was evaluated (Fig. S4b). On days 1 and 5, there was no substantial difference in the proliferation of BMSCs among the groups, whereas on day 3, the CaP/HS and MnCaP/HS groups showed higher proliferation than the remaining groups. At days 3 and 5, the proliferation of chondrocytes was better in the CaP/HS and MnCaP/HS groups than in the remaining groups, implying that the solid-liquid interface lubricating hydrogels had a promoting effect on chondrocyte proliferation. In addition, there was no difference in the cell viability of Raw264.7 cells among all groups. In conclusion, the solid-liquid interface lubricating hydrogels had good biocompatibility and met the needs of subsequent experiments targeting BMSCs, chondrocytes, and Raw264.7 cells as well as normal in vivo implantation.

### In vitro immunomodulation of solid-liquid interface lubricating hydrogels

**Fig. 3.**
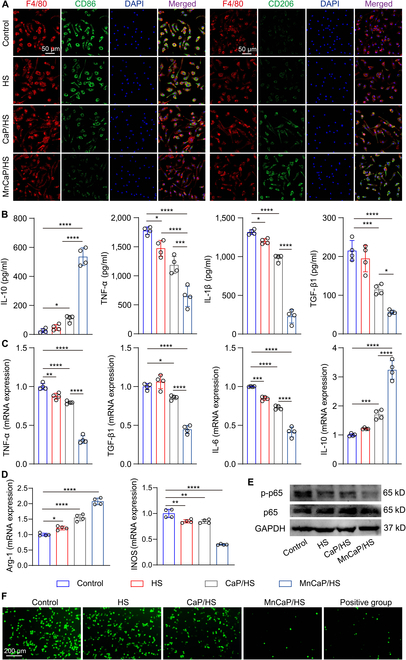
In vitro macrophage modulation by solid-liquid interface lubricating hydrogels. (A) Immunofluorescence staining of CD86 and CD206 after treatment of Raw264.7 cells with different hydrogels and (B) detection of secreted cytokine levels in the supernatant. Changes in the expression levels of (C) tumor necrosis factor-α (TNF-α), transforming growth factor-β1 (TGF-β1), interleukin-6 (IL-6), and interleukin-10 (IL-10) genes and (D) arginase 1 (Arg-1) and inducible nitric oxide synthase (INOS) genes in Raw264.7 cells treated with different hydrogels. Expression of (E) p65, p-p65, and (F) reactive oxygen species in Raw264.7 cells treated with different hydrogels (ns, no significant difference; *P < 0.05; **P < 0.005; ***P < 0.001; ****P < 0.0001). HS, HAMA/SA hydrogel; DAPI, 4′,6-diamidino-2-phenylindole; mRNA, messenger RNA; GAPDH, glyceraldehyde-3-phosphate dehydrogenase.

In an injured TB microenvironment, the immune cell inflammatory response triggered by prolonged frictional wear and stress concentration is an important cause of impeding cell differentiation and inducing tissue fibrosis, in which macrophages play an important role [37]. To assess how bioactive components in the solid-liquid interface lubricating hydrogels influence macrophage phenotype and their metabolism, CD86 and CD206 expression was examined using immunofluorescence staining. Compared with the control and HS groups, the CaP/HS- and MnCaP/HS-treated macrophages showed less expression of CD86 and more expression of CD206. This implied a higher degree of M2 polarization, and there was a notable difference between the MnCaP/HS group and the remaining groups (Fig. 3A and Fig. S5). In addition, we collected the supernatant after co-culture and determined the cytokines secreted by macrophages by enzyme immunoassay. As shown in Fig. 3B, the MnCaP/HS-treated macrophages secreted less tumor necrosis factor-α (TNF-α), interleukin-1β (IL-1β), and transforming growth factor-β1 (TGF-β1) and more interleukin-10 (IL-10), which suggested that manganese calcium phosphate enabled macrophages to secrete fewer pro-inflammatory cytokines and more anti-inflammatory cytokines, thereby balancing the inflammatory microenvironment. Studies have shown that overloaded mechanical stress is a major factor in the development of inflammation at the TB interface, while long-term oversecretion of inflammatory factors hinders TB healing. This can lead to the transformation of fibrocartilage regions at the TB interface into bone, as well as the formation of large amounts of scar tissue [38], thus failing to rebuild the TB microstructure and also reducing its biomechanical properties [39,40]. Interestingly, macrophages in the HS-treated group secreted less TNF-α and IL-1β compared to the control group, which might be due to the interaction of hyaluronic acid with macrophage surface receptors [41]. Qadri et al. [42] reported that hyaluronic acid reduced pro-inflammatory cytokine secretion through a CD44-mediated TLR2 inhibitory effect in macrophages. The changes in the relevant genes were further assessed by quantitative real-time polymerase chain reaction (q-PCR), and the results showed that the expression levels of TNF-α, TGF-β1, interleukin-6 (IL-6), and inducible nitric oxide synthase (INOS) genes were reduced in MnCaP/HS-treated macrophages, while the expression levels of arginase 1 (Arg-1) and IL-10 genes were enhanced, which is closely related to the regulation of macrophages by manganese ions (Fig. 3C and D). Western blot results showed that HS and MnCaP jointly reduced nuclear factor kappa-B (NF-κB) phosphorylation, suggesting that the solid-liquid interface lubricating hydrogel exerts anti-inflammatory effects by inhibiting the NF-κB pathway (Fig. 3E). Under oxidative stress conditions, we found that the MnCaP/HS group was able to reduce reactive oxygen species expression in macrophages more rapidly, which was attributed to its high expression of SOD (Fig. 3F and Fig. S6). Yang et al. [30] reported that active manganese sites, which simulated the coordination environment of metal sites in natural SOD and catalase, promoted the conversion of macrophages M1 to M2 through antioxidant effects. Xiong et al. [43] constructed a hollow-structured manganese-based nanoenzyme, which could inhibit oxidative damage through protection of mitochondrial functions and down-regulation of hypoxia inducible factor-1α (HIF-1α) expression and alleviate hypoxia, thereby modulating the transformation of the M1 to the M2 phenotype. Here, our designed solid-liquid interface lubricating hydrogels provided a favorable anti-inflammatory microenvironment for TB healing through hyaluronic acid.

### Solid-liquid interface lubricating hydrogels promote the chondrogenic differentiation of BMSCs

**Fig. 4.**
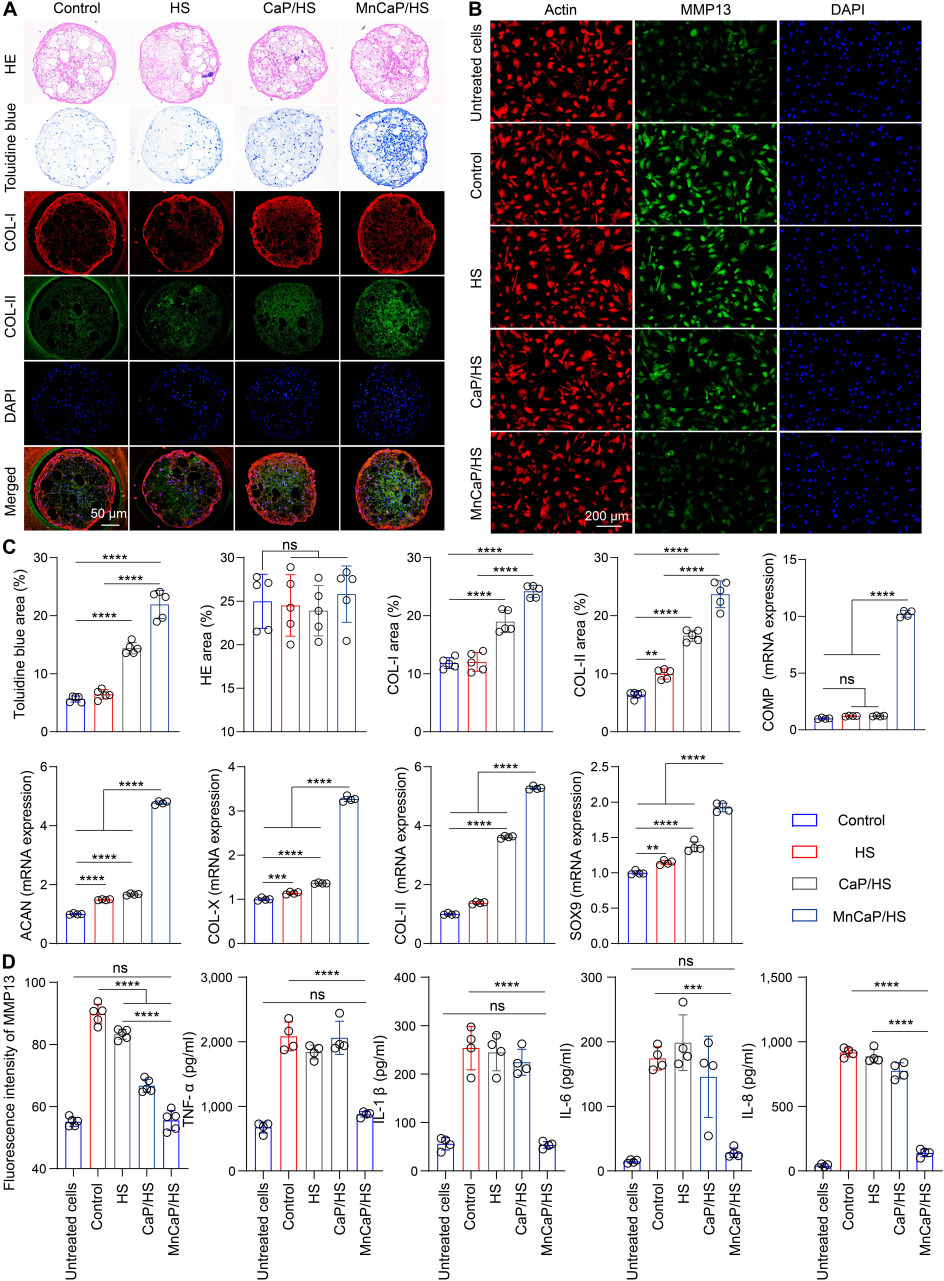
In vitro chondrogenic differentiation properties of solid-liquid interface lubricating hydrogels and their ability to protect chondrocytes under oxidative stress conditions. (A) Hematoxylin–eosin (HE), toluidine blue, and collagen I (COL-I)/collagen II (COL-II) staining after treating bone marrow mesenchymal stem cell (BMSC) spheres with different samples. (B) Immunofluorescence staining of matrix metallopeptidase 13 (MMP13) after treatment of chondrocytes with different samples under oxidative stress conditions. (C) ImageJ quantified the area of HE, toluidine blue, and COL-I/COL-II staining of BMSC spheres, and quantitative real-time polymerase chain reaction (q-PCR) detected the changes in the expression levels of chondrocyte-associated genes after different samples had been treated with BMSC spheres. (D) Secretion levels of cytokines after chondrocytes were treated with different samples (ns, no significant difference; **P < 0.005; ***P < 0.001; ****P < 0.0001). COMP, cartilage oligomeric matrix protein; ACAN, aggrecan; COL-X, collagen X; SOX9, SRY-box transcription factor 9.

The TB interface is a continuous tendon-chondro-bone structure, and after rupture of the TB interface, it is necessary to open the bone tunnel and connect the tendon to the bone by sutures [44], while promoting osteogenic and chondrogenic differentiation of BMSCs at the TB interface is crucial to re-establish a continuous tendon-chondro-bone structure [45]. Therefore, it is crucial to simulate and evaluate the effects of the bioactive components in the solid-liquid interface lubricating hydrogels on the chondrogenic differentiation of BMSCs in vitro. BMSC spheres were prepared by using low-adhesion well plates; treated sequentially with HS, CaP/HS, and MnCaP/HS extracts for 7 d; and stained with hematoxylin–eosin (HE) and toluidine blue (an alkaline positively charged dye that binds to chondroitin sulfate and hyaluronic acid in the extracellular matrix of cartilage). As shown in Fig. 4A, the control and HS groups, CaP/HS and MnCaP/HS groups had morphologically and structurally intact cytospheres, and the cells secreted a large amount of extracellular matrix. The MnCaP/HS-treated group showed a darker staining to toluidine blue, which implied a composition closer to the cartilage extracellular matrix. Further, we evaluated the extracellular matrix composition by collagen I (COL-I)/collagen II (COL-II) double labeling, and the MnCaP/HS-treated group secreted higher COL-I and COL-II than the control, HS, and CaP/HS groups. The quantitative analysis results confirmed this trend, which suggested that elemental Mn had an excellent osteogenic and chondrogenic role. Interestingly, the HS group did not show a marked osteogenic effect but could promote chondrogenic differentiation of BMSCs, whereas the CaP group exhibited a favorable action on both osteogenic and chondrogenic differentiation of BMSCs. Real-time quantitative polymerase chain reaction (RT-qPCR) results showed higher expression of the cartilage oligomeric matrix protein (COMP), SRY-box transcription factor 9 (SOX9), aggrecan (ACAN), collagen X (COL-X), and COL-II genes in the MnCaP/HS-treated group than those in the control, HS, and CaP/HS groups, which was consistent with the toluidine blue and COL-I/COL-II immunofluorescence staining results (Fig. 4C). Mn ions have been shown to promote proliferation and chondrogenic differentiation of BMSCs and can be applied to tissue-engineered scaffolds for the reconstruction of the cartilage-bone interface [31,46]. In addition, manganese-based grafts were reported to up-regulate SOX9, integrin, and COL-II expression in chondrocytes through the signaling pathways of p-Akt and p-ERK1/2 and maintained the cartilage phenotype at the site of injury [47]. Here, the MnCaP/HS group promoted chondrogenic differentiation of BMSCs in vitro, providing an experimental basis for cartilage formation in TB interface repair in vivo.

### Solid-liquid interface lubricating hydrogels protect chondrocytes under oxidative stress conditions

Following a TB interface tear, sustained motion and frictional wear induce oxidative stress in local tissues, which is detrimental to chondrogenic differentiation and exacerbates chondrocyte damage in BMSCs. Therefore, we used H_2_O_2_ to simulate oxidative stress conditions, and the experimental groupings were the untreated cell, control, HS, CaP/HS, and MnCaP/HS groups. We explored the protective effects of the bioactive components in the solid-liquid interface lubricating hydrogels on chondrocytes under oxidative stress conditions in vitro. As shown in Fig. 4B, the matrix metallopeptidase 13 (MMP13) expression in the MnCaP/HS group was lower than those in the remaining groups and comparable to that in the untreated control cell group. The fluorescence intensity data confirmed this trend, which indicated that MnCaP/HS could reduce H_2_O_2_ damage to chondrocytes. The level of inflammatory factor secretion by chondrocytes was further evaluated by enzyme immunoassay (Fig. 4D), and the results revealed that the MnCaP/HS-treated group secreted the least amount of TNF-α, IL-1β, IL-6, and IL-8, whereas the concentrations of TNF-α, IL-1β, and IL-6 were comparable to those of the untreated control cell group. This implied that the MnCaP/HS group ameliorated the oxidative stress state of chondrocytes and restored their normal metabolic levels, which also depended on manganese’s SOD-like and catalase-like activities [48]. Overall, the MnCaP/HS group protected chondrocytes from oxidative stress injury in vitro and favored cartilage formation and cartilage phenotype maintenance in TB interface repair in vivo.

### Solid-liquid interface lubricating hydrogels promote the osteogenic differentiation of BMSCs

**Fig. 5.**
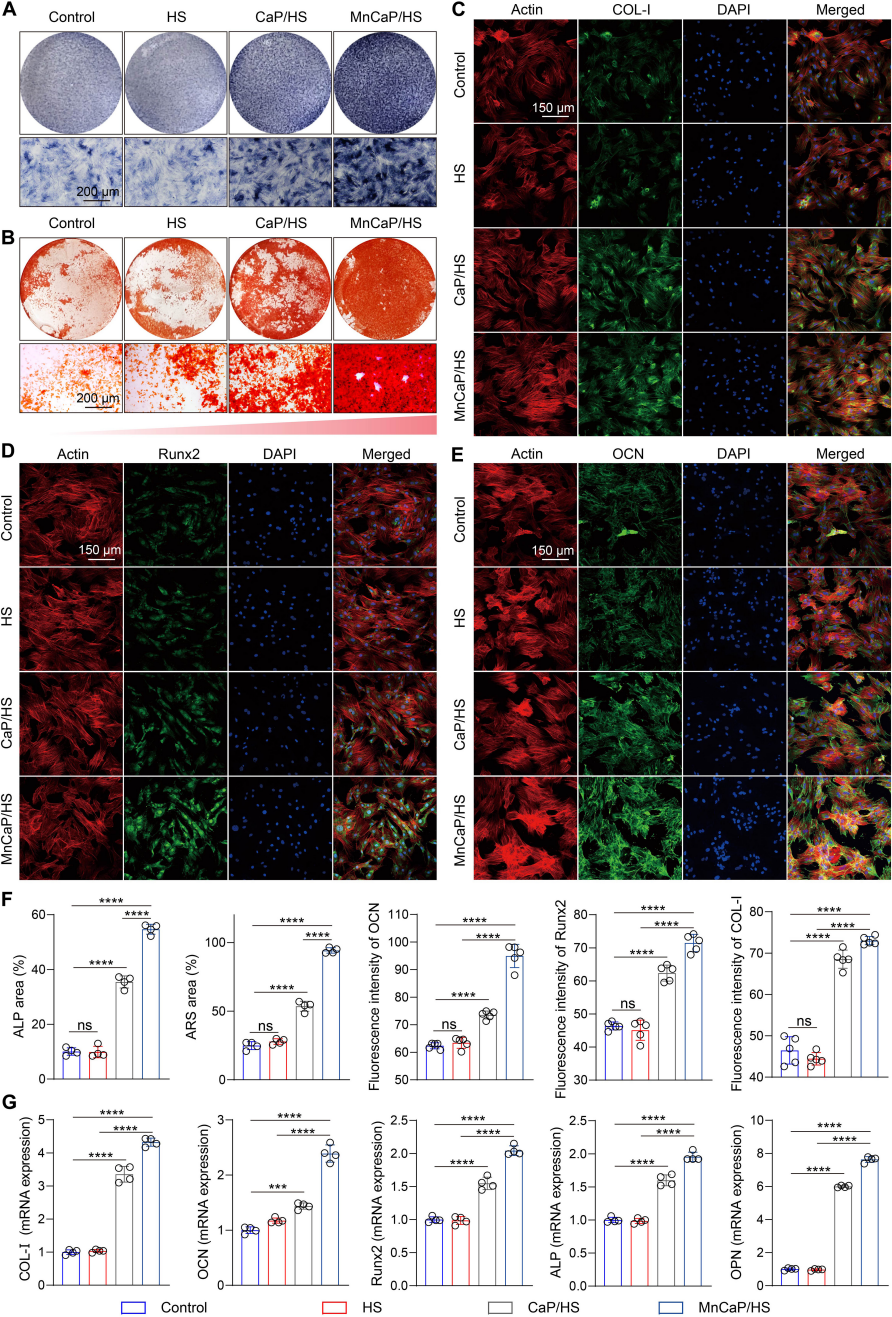
In vitro osteogenic differentiation properties of solid-liquid interface lubricating hydrogels. (A) Alkaline phosphatase (ALP) staining and (B) alizarin red S (ARS) staining images after treatment of BMSCs with different samples. (C) COL-I, (D) runt-related transcription factor 2 (Runx2), and (E) osteocalcin (OCN) immunofluorescence staining after BMSCs were treated with different samples. (F) Quantitative statistical plots of ALP, ARS, COL-I, Runx2, and OCN staining images. (G) q-PCR to detect the changes in the expression levels of osteogenesis-related genes after treatment of BMSCs with different samples (ns, no significant difference; ***P < 0.001; ****P < 0.0001). OPN, osteopontin.

The effective osteogenic differentiation of BMSCs is essential for the reconstruction of the TB interface, as it facilitates the establishment of tendon-chondrocyte-bone gradient structures and improves the mechanical properties of fragile tissues [49]. Therefore, we prepared conditioned media for macrophages and co-cultured them with BMSCs. Alkaline phosphatase (ALP) and alizarin red staining were performed to evaluate the early and late osteogenic differentiation status of BMSCs, respectively. As shown in Fig. 5A and B, more ALP and calcium nodules could be observed expressed in the MnCaP/HS-treated group compared with those in the control cell and HS- or CaP/HS-treated groups, confirming better osteogenic properties. Immunofluorescence staining was used to assess the COL-I, runt-related transcription factor 2 (Runx2), and osteocalcin (OCN) expression levels in BMSCs. MnCaP/HS-treated BMSCs expressed more COL-I and OCN in the extracellular matrix and more Runx2 in the nucleus than the other groups, and the fluorescence intensity also confirmed that more osteogenesis-related proteins were expressed in the MnCaP/HS group (Fig. 5C to F). RT-qPCR results showed that the CaP/HS and MnCaP/HS groups significantly up-regulated the expression levels of osteogenesis-related genes (COL-I, OCN, Runx2, ALP, and osteopontin [OPN]) as compared to the remaining 2 groups, which was attributed to the contribution of CaP to osteogenic differentiation. Meanwhile, the best osteogenic performance was found in the MnCaP/HS-treated group, implying that the immune microenvironment mediated by Mn ions further enhanced the osteogenic differentiation ability of BMSCs (Fig. 5G).

### In vivo evaluation of solid-liquid interface lubricating hydrogels for TB healing

**Fig. 6.**
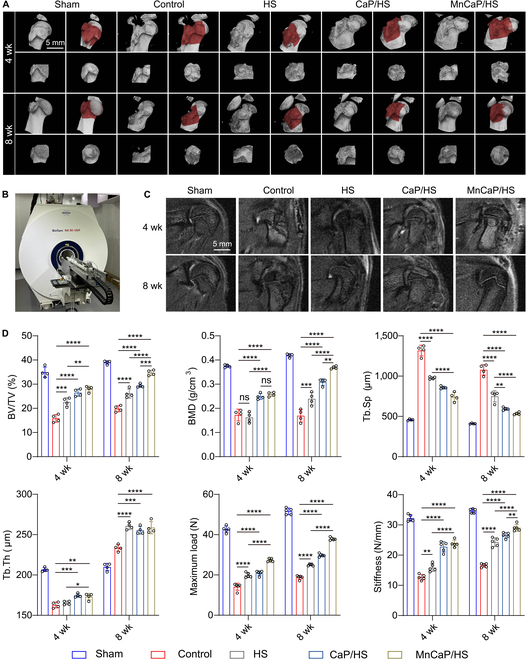
Solid-liquid interface lubricating hydrogels promote tendon-bone (TB) interface healing. (A) Three-dimensional (3D) reconstruction and (B and C) MRI pictures of TB sites at 4 and 8 weeks in different treatment groups. (D) Bone quality analysis and biomechanical analysis of the TB site at 4 and 8 weeks (ns, no significant difference; **P < 0.005; ***P < 0.001; ****P < 0.0001). BV/TV, trabecular volume fraction; BMD, bone mineral density; Tb.Sp, trabecular separation; Tb.Th, trabecular thickness.

Based on the above in vitro results, we further established a rat TB tear model to evaluate the effect of long-acting lubricating hydrogels on TB healing in vivo. The evaluations included imaging, mechanical, and histological evaluations combined with quantitative and qualitative results to assess the status of TB healing. At 4 and 8 weeks, hard-tissue reconstruction and soft-tissue assessment of the TB injury site in rats were performed using micro-computed tomography (micro-CT) and MRI in the rats, respectively. The micro-CT results showed that at 4 and 8 weeks, the TB structure was intact in the sham group (unoperated healthy rats) and severely damaged in the control group (PBS-treated). The lack of durable lubrication resulted in stress concentrations and limited shoulder motion. Compared with that in the control group, the TB interface structure was more intact in the HS group at 4 and 8 weeks. This was attributed to the fact that HS provided a continuous solid interface and a solid-liquid interface lubrication microenvironment, which ameliorated the stress concentration induced by excessive friction at the TB interface during exercise. However, the structure was still not completely repaired at 8 weeks, suggesting that long-lasting lubricating properties alone were not enough for promoting healing. Compared to the HS group, the CaP/HS group showed better TB healing, but its tough tissue structure at 8 weeks indicated that healing was still not complete. Importantly, the MnCaP/HS group showed better healing at 4 weeks as compared to the other groups, and the TB interface was healed at 8 weeks (Fig. 6A). MRI was more favorable for observing soft-tissue healing at the TB interface, and a stronger contrast of the images between the tissues in the MnCaP/HS-treated group at 4 weeks was observed, which might be due to the MRI enhancement effect of manganese ions (Fig. 6B and C). Quantitative data were analyzed for bone mineral density, trabecular thickness, trabecular separation, and trabecular volume fraction in each group, and the results demonstrated faster osteogenesis and better bone quality in the MnCaP/HS group. In addition to the imaging data, we further evaluated the effect of the solid-liquid interface lubricating hydrogels on TB interface healing by biomechanical tests to assess the maximum load and stiffness of the TB at 4 and 8 weeks postoperatively. The biomechanical properties of TB in the sham group were the best, and those of TB in the HS group were better than those in the control group, with a statistically significant difference. This suggests that solid-liquid interface lubricating hydrogels could extremely improve the healing quality of the TB interface by providing continuous solid lubrication, and solid-liquid lubrication could improve the healing quality of the TB interface. Among all experimental groups, the MnCaP/HS group showed the highest maximum load and stiffness, further confirming that MnCaP maximally promoted high-quality healing of the TB interface under the effect of long-lasting lubrication (Fig. 6D).

**Fig. 7.**
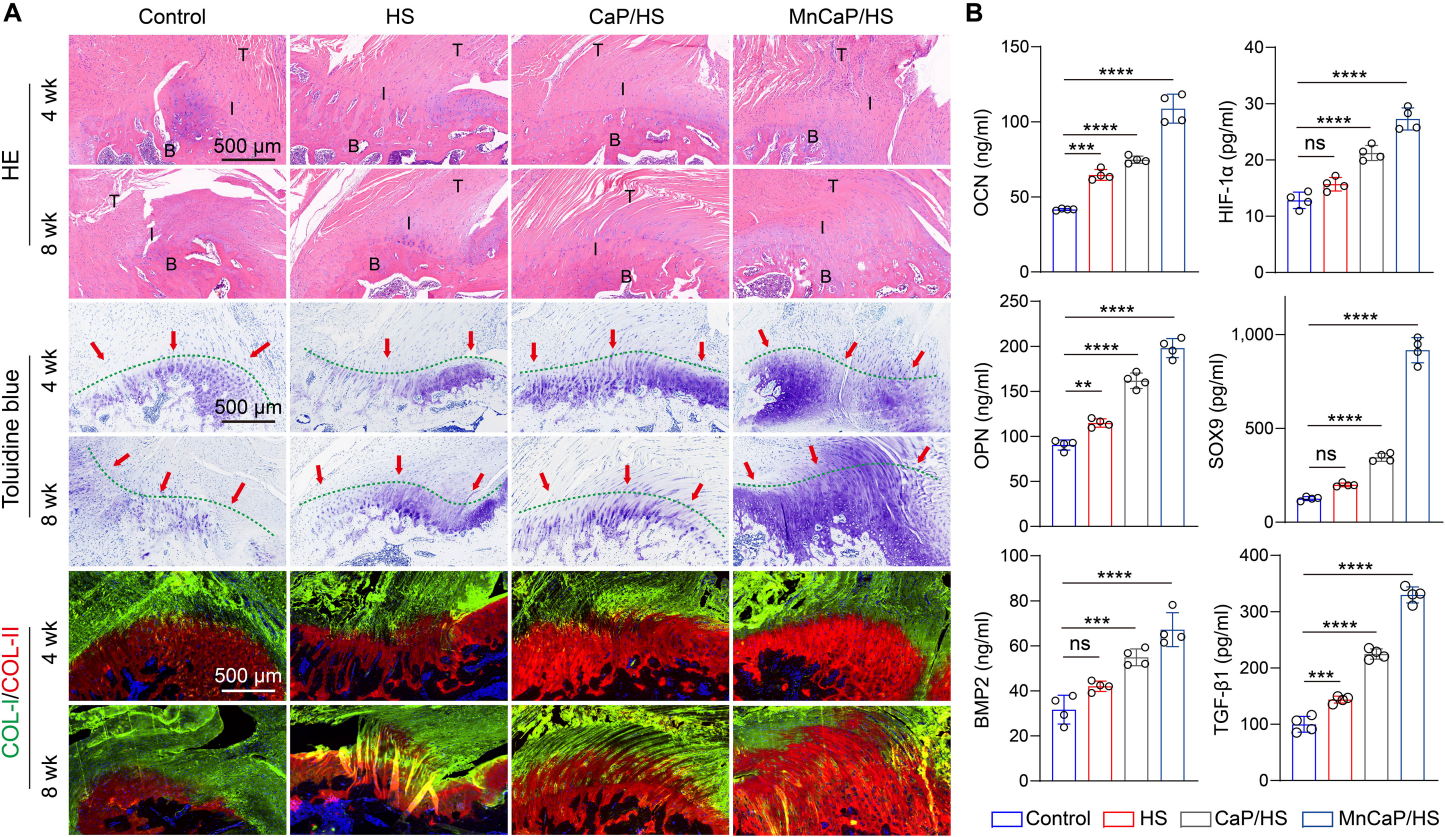
Histological evaluation of solid-liquid interface lubricating hydrogels for promoting the healing of the TB interface. (A) HE, toluidine blue, and COL-I/COL-II staining of TB sites in different treatment groups at 4 and 8 weeks. (B) Secretion levels of osteogenesis- and chondrogenesis-related cytokines at TB sites in different treatment groups at 4 weeks (ns, no significant difference; **P < 0.005; ***P < 0.001; ****P < 0.0001). T, tendon; I, interface; B, bone; HIF-1α, hypoxia inducible factor-1α; BMP2, bone morphogenetic protein 2.

**Fig. 8.**
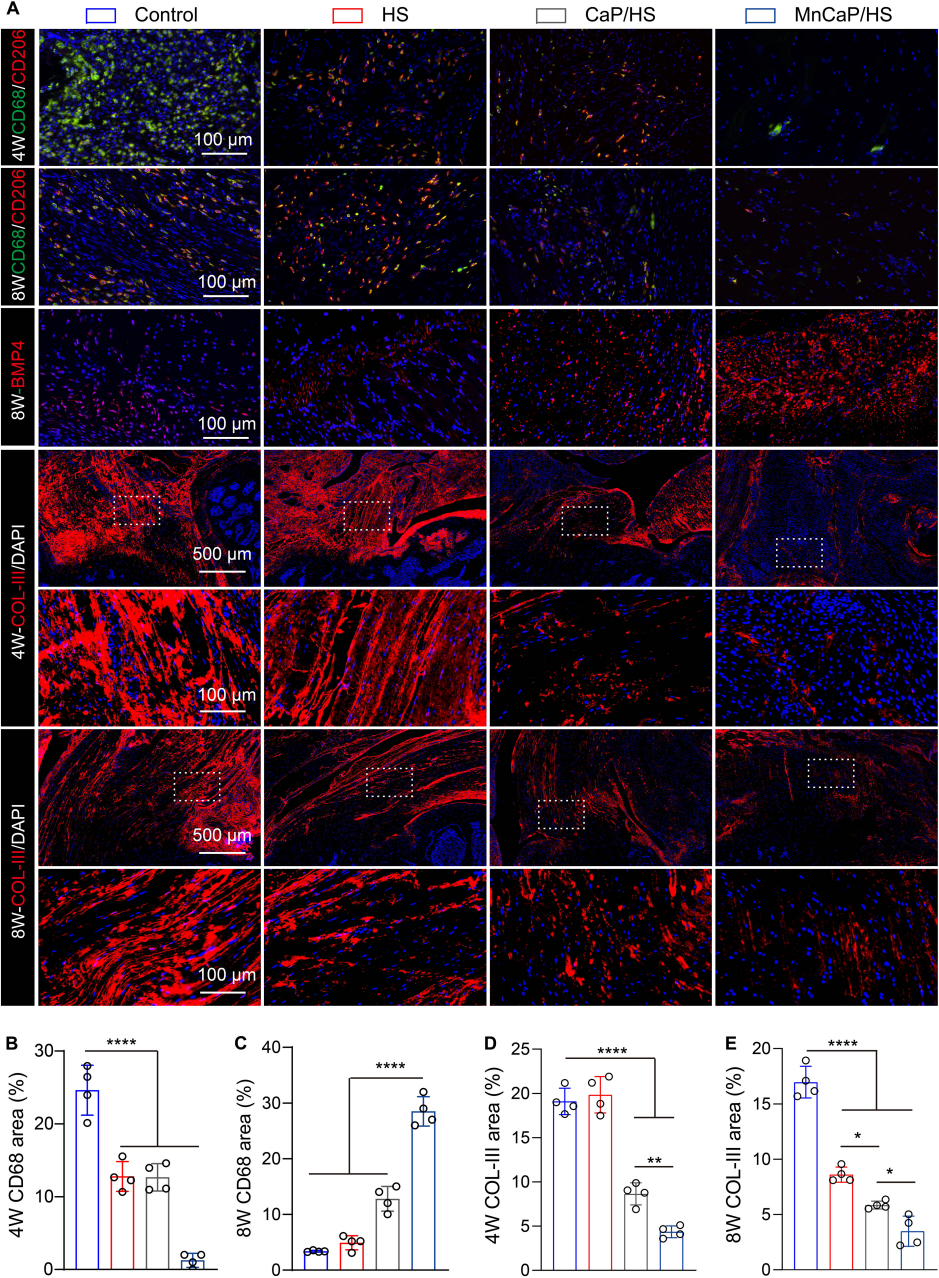
Immunomodulation and scar formation of solid-liquid interface lubricating hydrogels at the TB interface. Quantitative analysis of (A) CD68/CD206 staining, BMP4 staining, and collagen III (COL-III) staining and their (B to E) areas at TB sites at 4 and 8 weeks in different treatment groups (*P < 0.05; **P < 0.005; ****P < 0.0001). 4W, 4 weeks; 8W, 8 weeks.

In fact, the mechanical cushioning capacity of the injured TB interface is drastically reduced, and stress concentration and frictional wear lead to the long-term accumulation of pro-inflammatory M1 macrophages. This results in the development of chronic inflammation at the TB interface, and a large amount of inflammatory factors accelerates bone turnover and inhibits the regeneration of the fibrocartilage layer [50]. Current research still focuses on the use of bioactive substances to reduce the inflammatory response and promote the regeneration of the TB interface. Solid-liquid interface lubricating hydrogels have not yet been reported for relieving stress concentration and frictional wear, and their biological effects at the TB interface have not yet been effectively validated. Therefore, we examined the concentration of inflammatory factors (TNF-α, IL-6, and IL-1β) at the TB injury site at 3 and 7 d postoperatively to assess the early inflammatory response (Fig. S7). Data on the heart, liver, spleen, lungs, and kidneys at 8 weeks showed that none of the implanted materials in any group exhibited long-term toxicity (Fig. S8). Compared with that in the control group, the concentration of inflammatory factors decreased in the remaining groups at 3 and 7 d, implying that the implantation of the solid-liquid interface lubricating hydrogels improved the early inflammatory response at the TB site. Moreover, the level of inflammatory factors in the MnCaP/HS group was lower than that in the HS group, further confirming that the anti-inflammatory effect of HS was enhanced by MnCaP. To assess the effects of the long-lasting lubrication microenvironment and anti-inflammatory microenvironment on the regeneration of the TB interface, the tissue slides were histologically analyzed with HE and toluidine blue staining. The HE results showed that the collagen fibers in the control group were irregular, whereas the collagen fibers in the HS, CaP/HS, and MnCaP/HS groups were more ordered in their distribution. This demonstrated a better tendon-cartilage-bone gradient structure, indicating that the solid-liquid interface lubricating hydrogels improved the regenerative microenvironment and microstructural reconstruction of the TB interface. Toluidine blue staining showed less neoplastic fibrocartilage in the control group and more neoplastic fibrocartilage in the MnCaP/HS group, suggesting that an ordered tendon-chondrocyte-bone gradient structure had been formed. COL-I/COL-II immunofluorescence staining demonstrated a stable tendon-chondrocyte transition layer in the MnCaP/HS group at both 4 and 8 weeks, whereas the control group’s cartilage layer decreased at 8 weeks, which might be caused by the absence of solid-liquid interface lubricating hydrogels to relieve stress concentration and frictional wear (Fig. 7A). In addition, we examined the expression of osteogenesis/chondrogenesis-related cytokines in the TB tissues and compared it with that in the control group. The long-acting solid-liquid interface lubricating hydrogels treated groups secreted more OPN, OCN, and TGF-β1, and the MnCaP/HS-treated group also promoted the expression of HIF-1α and SOX9, which contributed to the reconstruction of the cartilage-bone microstructure (Fig. 7B). Chronic inflammation and scar formation during TB healing were assessed by immunofluorescence staining (Fig. 8A). CD68/CD206 immunofluorescence pictures at 4 and 8 weeks showed that many macrophages were present at the TB interface for a long period of time in the control group. This indicated that there was a chronic inflammatory response at this site. Meanwhile, the remaining groups showed a significant decrease in the expression of CD68, suggesting that the TB was exposed with lowered chronic inflammatory response and shifted from the inflammatory phase to the healing phase. BMP4 immunofluorescence showed that group A expressed higher levels of BMP4 protein than other groups. Again, the best results were found in the MnCaP/HS group. Collagen III (COL-III) secreted by fibroblasts is the main component of scar tissue, and reducing COL-III deposition in the extracellular matrix is the key to inhibiting scar formation and restoring the mechanical properties of TB [51]. At 4 weeks, both the control and HS groups showed more COL-III deposition, and the CaP/HS and MnCaP/HS groups demonstrated less COL-III deposition. At 8 weeks, COL-III deposition was significantly decreased in the remaining groups compared to that in the control group, suggesting that the chronically lubricated microenvironment improved the regenerative microenvironment of the TB interface and reduced scar formation. The MnCaP/HS group had the least amount of COL-III deposition, which was related to the biological function of MnCaP (Fig. 8B to E). Meanwhile, this trend was consistent with the biomechanical properties of TB after treatment in all groups, and the reduced scar tissue improved the mechanical properties of TB.

### Transcriptome analysis of solid-liquid interface lubricating hydrogels for TB healing

**Fig. 9.**
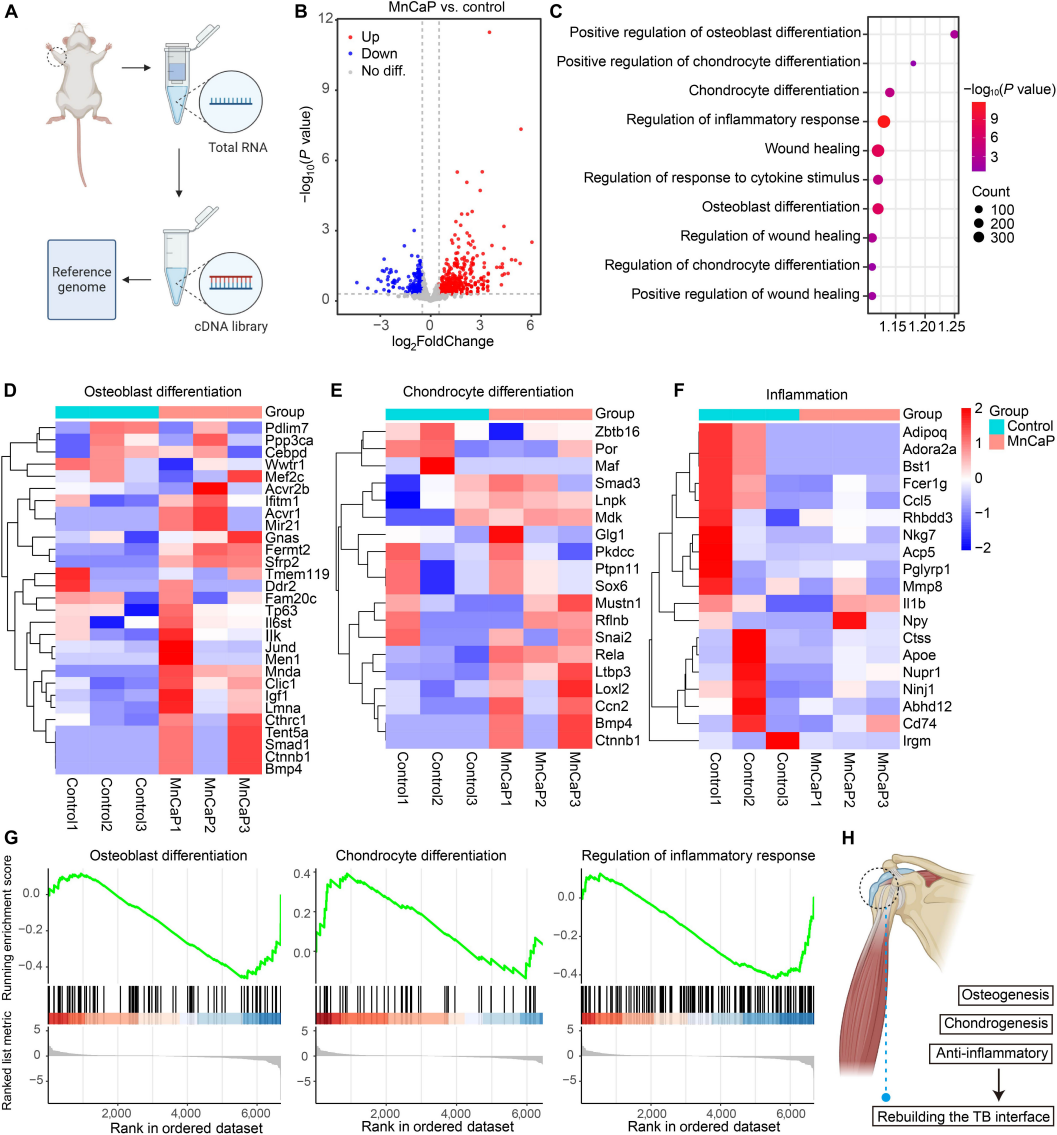
Transcriptomic analysis of solid-liquid interface lubricating hydrogels’ repair of the TB interface. (A) TB interface sample collection for transcriptomic analysis. Significant differential genes between the MnCaP/HS group and the control group in the (B) volcano map, (C) Gene Ontology (GO) enrichment analysis, (D to F) hierarchical clustering heat map, and (G) gene set enrichment analysis (GSEA) results. (H) Schematic diagram of TB interface healing. cDNA, complementary DNA.

**Fig. 10.**
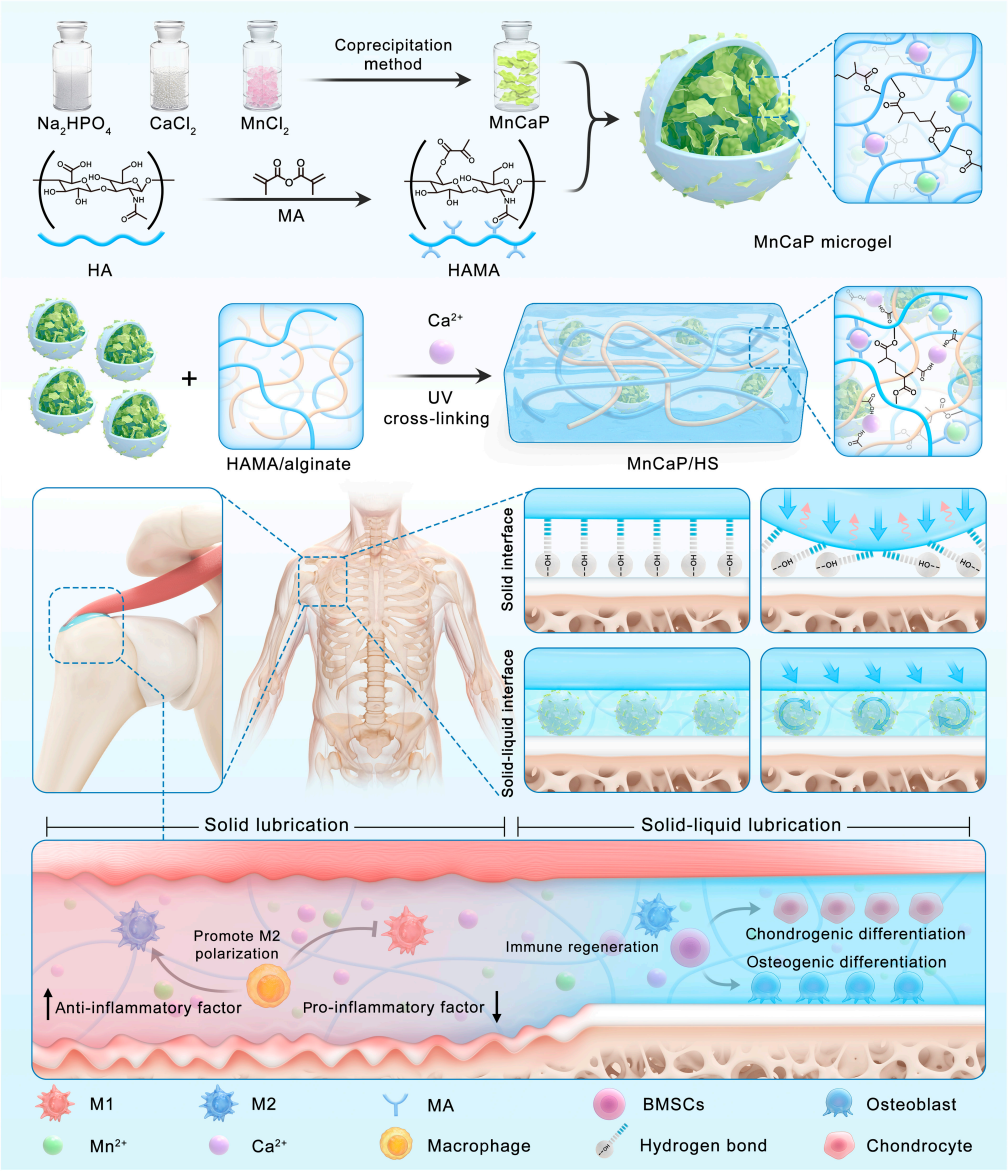
Solid-liquid interface lubricating hydrogels for TB healing. Hydrogels resist stress damage through surface hydrogen bonds and ionic toughening, while microgels and degradation products achieve long-lasting lubrication through rolling lubrication layers and liquid lubrication films, respectively. HA, hyaluronic acid; MA, methacrylic anhydride.

Rat TB specimens were isolated 8 weeks after surgery, and RNA sequencing analysis was performed on the control and MnCaP/HS groups (Fig. 9A), and the screening criteria for this analysis were *P* value < 0.05 and |log_2_FoldChange| > 0.5. As shown in the figure, the differential genes of MnCaP/HS vs. control included 233 up-regulated genes and 362 down-regulated genes (Fig. 9B). Gene Ontology analysis showed that these differential genes were associated with osteogenic differentiation, chondrogenic differentiation, inflammatory response, and cytokine secretion, and these genes were also actively involved in tissue repair processes (Fig. 9C). Among them, genes related to osteogenic differentiation, such as Smad1, BMP4, and Sfrp2, were up-regulated, suggesting that solid-liquid interface lubricating hydrogels may promote osteogenesis at the TB interface through the BMP4/Smad1 pathway [52]; genes related to chondrogenic differentiation, such as Sox6, Smad3, and Snai2, were also up-regulated; and genes related to inflammatory response, such as MMP8, CD74, and Ccl5, were down-regulated, which suggested that the MnCaP/HS hydrogel inhibited inflammatory response occurrence at the TB interface and actively promoted bone-cartilage microstructural reconstruction (Fig. 9D to F). To further investigate the alterations in signaling pathways between the control and MnCaP/HS groups, we analyzed the weighted scores of signaling pathways related to osteogenic differentiation, chondrogenic differentiation, and inflammatory response using gene set enrichment analysis (Fig. 9G), and the results showed that signaling pathways associated with some osteogenic and chondrogenic genes were up-regulated, and those associated with inflammation were down-regulated, which implied that the MnCaP/HS played a vital role in contributing to bone/chondral repair and anti-inflammation at the TB interface (Fig. 9H). Gene set enrichment analysis showed that the Wnt signaling pathway and NF-κB signaling pathway were coordinately enriched in differentially expressed genes, and the core gene interaction network of osteogenesis, chondrogenesis, and inflammation also confirmed this conclusion (Fig. S9a to d). In summary, this paper proposes a solid-liquid interface lubricating hydrogel that promotes TB healing through a “sustained lubrication-immune regeneration coupling” mechanism (Fig. 10). First, the energy dissipation of solid-liquid interface lubricating hydrogel reduces local stress-induced microdamage to provide sustained lubrication, which inhibits the activation of the NF-κB pathway and down-regulates pro-inflammatory factors. Mn ions further promote the transformation of macrophages from M1 to M2 and the secretion of anti-inflammatory factors while also promoting the osteogenic/chondrogenic differentiation of BMSCs. This synergistically improves the immune regeneration microenvironment of TB, forming a positive feedback loop of “low inflammation–high-efficiency repair”.

In conclusion, although lubricating biomaterials have demonstrated advantages in reducing inflammation, decreasing tendon adhesions, and relieving stress concentrations, they have been less reported in the field of TB repair. Solid-liquid interface lubricating hydrogels have great potential for TB repair, as, on the one hand, they can provide continuous lubrication in the early postoperative phase and reduce slip resistance, thereby promoting collagen fiber orientation and improving mechanical durability. On the other hand, they can be effectively loaded with drugs, growth factors, and other bioactive substances, and the release rate of the lubricant can be precisely controlled to synergistically promote TB healing. To further enhance the application of solid-liquid interface lubricating hydrogels in TB repair, it is an important direction for future research to synchronously optimize the multilevel structure and composition ratio of solid-liquid interface lubricating hydrogels, which can balance lubrication performance and mechanical support; in addition, intelligent lubrication systems with responsive and self-repairing functions can be developed to achieve low immunogenicity and degradation rates that match the TB healing cycle, thereby efficiently repairing TB damage environments. Moreover, our “continuous lubrication–immune regeneration” strategy is primarily aimed at the early window of TB healing and interface integration. For chronic degeneration/high-tension/heavy-load athletes and other scenarios, further validation and optimization of loading and dosage are required in large-animal models (such as sheep, dogs, and pigs). Large-animal and preclinical studies to systematically evaluate the biosafety, degradation products, and long-term repair effects of solid-liquid interfacial lubrication hydrogels will also promote their clinical translation.

## Conclusion

In this study, solid-liquid interface lubricating hydrogels were prepared by first constructing a calcium manganese phosphate microgel by ionic coordination and then loading it into a hyaluronic acid/SA dual network by covalent/ionic cross-linking. The hydrogels ensure the long-term effectiveness of synergistic lubrication at the solid-liquid interface at TB injury sites and the synergistic regulatory effect of Mn and CaP on immune differentiation. Firstly, in the pre-TB healing stage, the hydrogels enhanced the solid lubrication interface through surface hydrogen bonding hydration and ionic bonding toughening, which shielded stress damage and improved the early inflammatory microenvironment. As the TB healed, the calcium manganese phosphate microgel was gradually released and formed a solid-liquid lubrication interface with the hydrogel degradation products. This provided continuous lubrication through the rolling lubrication layer and liquid lubrication film, alleviating the chronic inflammatory response and forming a favorable immune-regenerative microenvironment. In addition, CD68/CD206 and COL-I/COL-II staining in vivo confirmed that the metal ions released from the calcium manganese phosphate microgel improved the inflammatory microenvironment to further promote the cartilage-bone gradient differentiation of BMSCs and inhibit the formation of scar tissue, which ultimately achieved structural and functional repair. In conclusion, MnCaP/HS provides a promising preclinical basis for TB healing by providing a durable lubricating and regenerative microenvironment, with potential clinical value in reducing TB friction-related inflammation and promoting interface integration.

## Materials and Methods

### Materials

Hyaluronic acid (molecular weight = 200,000) was purchased from Huaxi, China; methacrylic anhydride (99%; product number: 4093), calcium chloride (97%; product number: 5284359), manganese chloride (98%; product number: 24845346), diammonium hydrogen phosphate (98.5%; product number: 24540), and ammonia (25% in H_2_O; product number: 14923) were purchased from Aladdin, Shanghai. SA (product number: 180947), lithium phenyl-2,4,6-trimethylbenzoylphosphinate (LAP, 95%; product number: 900889), and type II collagenase (product number: C2-BIOC) were purchased from Sigma-Aldrich, Germany.

### Preparation and characterization of solid-liquid interface lubricating hydrogels

The solid-liquid interface lubricating hydrogels consisted of calcium manganese phosphate microgel and hyaluronic acid/SA hydrogels. Firstly, 1.457 mM calcium chloride and 0.1619 mM manganese chloride solution (Ca:Mn = 9:1) were configured as liquid A and 0.977 mM ammonium dihydrogen phosphate as liquid B. MnCaP nanosheets were prepared by mixing liquids A and B by the coprecipitation method (Ca + Mn) and P in a molar ratio of 1.65:1. The entire reaction was carried out at 80 °C, with the pH maintained at 7. The precipitate was then collected by ultracentrifugation at 10,000 rpm, washed 3 times with ethanol, freeze-dried, and stored for later use. Methacrylated hyaluronic acid was prepared according to our previous literature [53–55]. Five milligrams of methacrylated hyaluronic acid, 2 mg of MnCaP, and 0.5 mg of LAP were dispersed in 1 ml of water, and a MnCaP microgel was formed by ultraviolet irradiation for 2 min (10 W, 365 nm). The physical phase, morphology, elemental distribution, elemental valence states, and MRI capability of the MnCaP nanosheets were characterized using XRD (2*θ* angle range 10° to 80°, step size 10°/min), SEM, TEM, XPS, and MRI, respectively. Optical microscopy, SEM, and Fourier transform infrared spectroscopy (400 to 2,500 cm^−1^, the resolution is 2 cm^−1^) were utilized to assess the particle size, morphology, elemental distribution, and chemical composition of MnCaP microgels.

The hyaluronic acid/SA dual-network hydrogel consisted of 2.5 wt% methacrylated hyaluronic acid, 2 wt% SA, MnCaP microgel (final concentration of 1 mg/ml of MnCaP), and 0.25 wt% LAP, and the final product was obtained by photo-cross-linking for 2 min (10 W, 365 nm) followed by immersion in a 2 wt% calcium chloride solution for 30 min (25 °C, 60 rpm shaking incubator). Pressure was slowly applied to the hydrogels at 50% deformation, and the deformation was observed and recorded. The morphology of hyaluronic acid/SA dual-network hydrogels was observed using SEM. The compression resistance and toughness of the hydrogels (⌀ 8 × 4 mm) were evaluated using an electronic universal testing machine (the loading rate is 1 mm/min, *n* = 3). The energy storage modulus and dissipation modulus of the hydrogels (⌀ 8 × 4 mm) were evaluated using a rheometer. The hydrogels were placed in PBS solution at 37 °C and degraded continuously on a shaker for 4 weeks and evaluated for weight loss. Calcium manganese phosphate microspheres were compounded with hyaluronic acid/SA bi-network hydrogels and subjected to degradation experiments in PBS solution at 37 °C. The degradation products were collected, and their friction coefficients were evaluated by a friction and wear tester (Bruker, Germany) at 1, 2, 3, and 4 weeks. Specifically, the friction pair consists of polytetrafluoroethylene and stainless-steel spheres (diameter of 5 mm) with a load of 15 N (25 °C, humidity below 80%). The reciprocating sliding distance amplitude and sliding frequency are 4 mm and 1 Hz, respectively. The release of ions during the degradation process was detected by an inductively coupled plasma emission spectrometer at 1, 3, 5, 7, 14, 21, and 28 d (PerkinElmer, USA).

### Biocompatibility

BMSCs were obtained by collecting bone marrow suspensions from 4-week-old male Sprague Dawley rat femur and tibia, and chondrocytes were obtained by isolating rat cartilage and digesting it with type II collagenase. BMSCs were collected using the adhesion method, and P3 to P6 generation cells were used in subsequent experiments. After co-incubation of each group of 50 mg of hydrogels with 1 ml of Dulbecco’s modified Eagle medium for 24 h (37 °C, shaking table frequency of 100 rpm), the supernatant was collected, and a conditioned medium was obtained by supplementing with 10% fetal bovine serum and 1% penicillin/streptomycin. The conditioned media were co-cultured with BMSCs, chondrocytes, and macrophages, respectively. Cell viability was evaluated by Cell Counting Kit-8 (Beyotime, China), and cell morphology was assessed by Actin-Tracker Red-555 and DAPI (Beyotime, China). The conditioned medium for culturing macrophages did not contain 1% penicillin/streptomycin.

### In vitro macrophage immunomodulatory capacity

After co-culture of hydrogels with macrophages, the macrophages were treated with CD86 and CD206 antibodies (Abcam, 1:1,000) for 12 h. The fluorescence was recorded by laser confocal microscopy (ZEISS, Germany), and fluorescence intensity was detected by ImageJ. The supernatants were collected to detect TNF-α, IL-1β, TGF-β1, and IL-10 (Chutai, China). PCR was conducted to analyze the levels of macrophage-related genes (TNF-α, TGF-β1, IL-6, and IL-10). Reactive oxygen species and SOD expression levels were evaluated after hydrogel incubation with macrophages under oxidative stress conditions (50 μM H_2_O_2_, 5 h).

### In vitro chondrogenic differentiation properties and ability to protect chondrocytes under oxidative stress conditions

BMSC spheres were prepared using ultralow adhesion plates and co-cultured for 7 d with the conditioned medium prepared above (supplemented with chondrogenic induction medium). Then, after fixation using 4% paraformaldehyde, they were embedded in agar and sectioned. The chondrogenic differentiation properties of BMSC spheres were assessed by HE staining, toluidine blue staining, and immunofluorescence staining (COL-I/COL-II). PCR was performed to detect the expression of genes related to chondrogenic differentiation (COMP, ACAN, COL-II, COL-X, and SOX9) in the cell spheres of BMSCs (the annealing temperature was 60 °C; the internal reference gene was GAPDH). To assess the protective effect of solid-liquid interface lubricating hydrogels on chondrocytes under oxidative stress conditions, 50 μM H_2_O_2_ and conditioned medium were used to co-incubate with chondrocytes for 12 h. The expression of MMP13 in chondrocytes was assessed by immunofluorescence staining, and the medium was collected and tested for the concentration of cytokines (TNF-α, IL-1β, IL-6, and IL-8).

### In vitro osteogenic differentiation properties

BMSCs were cultured with conditioned medium (including an equal proportion of osteogenic induction medium), and ALP and alizarin red S staining (Beyotime, China) were performed at 7 and 21 d, respectively. The above BMSCs were treated for 12 h with Runx2, OCN, and COL-I (Abcam, 1:1,000) antibodies, and fluorescence was recorded by laser confocal microscopy. The expression of osteogenesis-related genes in BMSCs was examined by PCR (ALP, Runx2, COL-I, OPN, and OCN).

### Animal experiments

#### Establishment of the TB tear model and implantation of solid-liquid interface lubricating hydrogels

All animal experiments were approved by the Animal Ethics Committee of Ruijin Hospital, Shanghai Jiaotong University (2023-02-SGKYJS-CWG-041), and the experimental animals were 220- to 250-g Sprague Dawley male rats, with groups of sham, control, HS, CaP/HS, and MnCaP/HS (*n* = 12). During surgery, rats were anesthetized with isoflurane gas, and after exposing the rotator cuff region of the rat, surgical sutures were used to cross the tendon at the TB and then completely incise the junction between the tendon and bone. In addition, a suture needle was used to pass through both sides of the bone and create 1-mm bone tunnels, the suture connected to the tendon was crossed through the bone tunnel, a solid-liquid interface lubricating hydrogel (4 × 3 × 1 mm) was implanted at the TB interface, the tendon was connected to the bone, and the wound was closed sequentially. After suturing the wound and disinfecting the wound, antibiotics were administered for 2 consecutive days without restricting the rats’ activity, and the rats were euthanized and sampled at 3 d, 7 d, 4 weeks, and 8 weeks, respectively. Of these, tissue samples at 3 and 7 d were used to detect inflammatory factors. The tissue removed after 8 weeks was stored in liquid nitrogen and subjected to standardized transcriptomic analysis, including strict quality control and removal of low-expression genes (*n* = 3).

### Micro-CT, MRI, and biomechanics were used to assess the quality of TB regeneration

A micro-CT scanner (SkyScan 1276, Germany) was used to evaluate TB in rats at 4 and 8 weeks. Three-dimensional bone tissue reconstruction images were obtained by mimics, and trabecular volume fraction, bone mineral density, trabecular separation, and trabecular thickness were analyzed. MRI (BioSpin, Germany) was used to assess the state of soft-tissue healing at the TB interface. Biomechanical tests (Instron 5969, USA) were used to assess the mechanical properties of the healed TB (*n* = 5). One end was fixed to the bone fracture, and the other end was fixed to the tendon. The stretching speed was 1 mm/min, and the maximum stretching force was recorded as the maximum load.

### Histological evaluation

TB tissues were fixed with 4% paraformaldehyde for 48 h, decalcified with 10% EDTA for 4 weeks, and then assessed for decalcification using the needle prick method. After completion, the tissue was embedded in paraffin. The slice thickness was 5 μm, followed by HE and toluidine blue staining, with toluidine blue staining for 25 min, separation using 95% ethanol, and finally dehydration and clearing. In addition, the expression levels of COL-I/COL-II, CD68, and COL-III in the tissues were analyzed by immunofluorescence staining and quantified by ImageJ (*n* = 4). Among them, the contour tracing method is used to select the region of interest area, and the background is subtracted by adjusting the threshold to calculate the positive area in the region of interest area.

### Statistical analysis

The experiment was repeated at least 3 times, and the results are expressed as mean ± standard deviation. The GraphPad Prism 9.0 software and Origin 2018 software were used for plotting and statistical analysis. Analysis of variance was employed to compare experimental data (ns, no significant difference; *P < 0.05; **P < 0.005; ***P < 0.001; ****P < 0.0001).

## Data Availability

Data will be made available on request.
