# Supplementary material for: Solid-Liquid Interface Lubricating Hydrogels for Tendon-Bone Healing
Source: Research (Wash D C). 2025 Oct 23;8:0924. doi: 10.34133/research.0924 (PMC12547148; doi:10.34133/research.0924)
Supplement: Supplementary 1 — Figs. S1 to S9 Table S1 [file research.0924.f1.docx]

**Solid-liquid interface lubricating hydrogels for tendon-bone healing**

Pengzhen Zhuang, Liang Chen , Yu Zhang, Wu Yang, Yu Chen, Guilai Zuo, Longxi Wu, Lei Xiang, Zhen Wang, Jessica M. Rosenholm, Tingjun Ye, Hongbo Zhang, Wenguo Cui


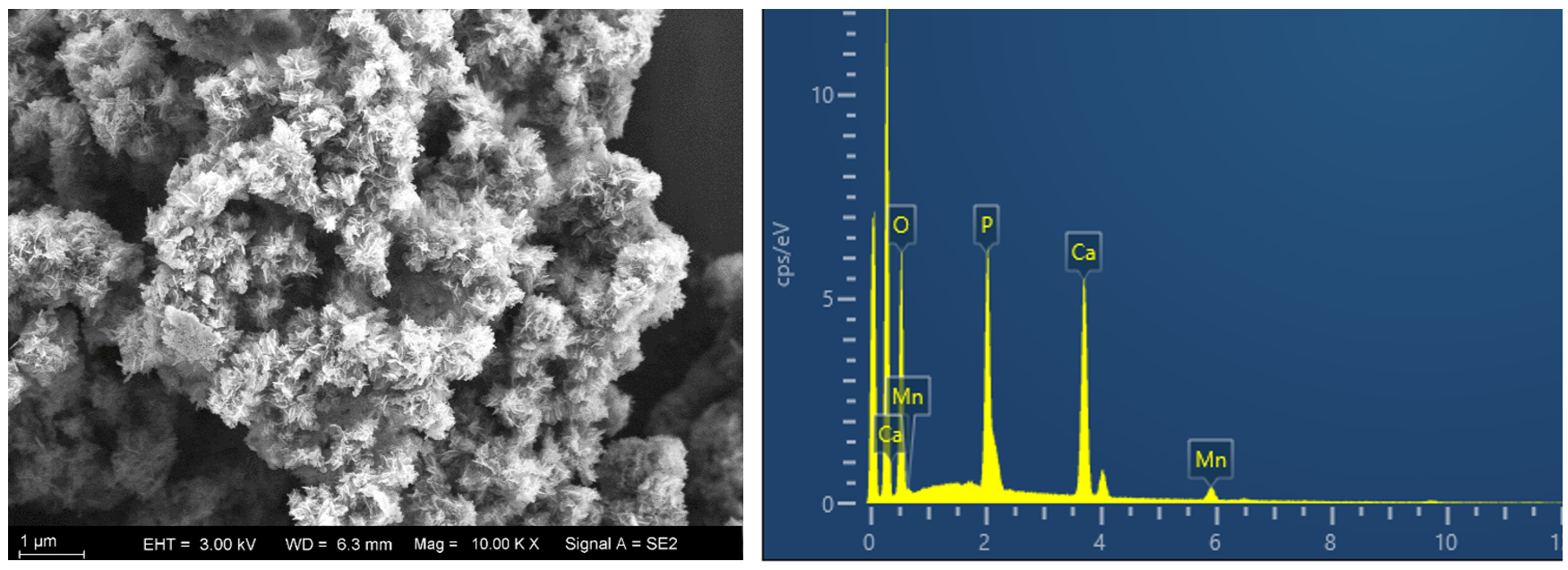


Figure S1 SEM images and elemental distribution profiles of calcium manganese phosphate nanosheets


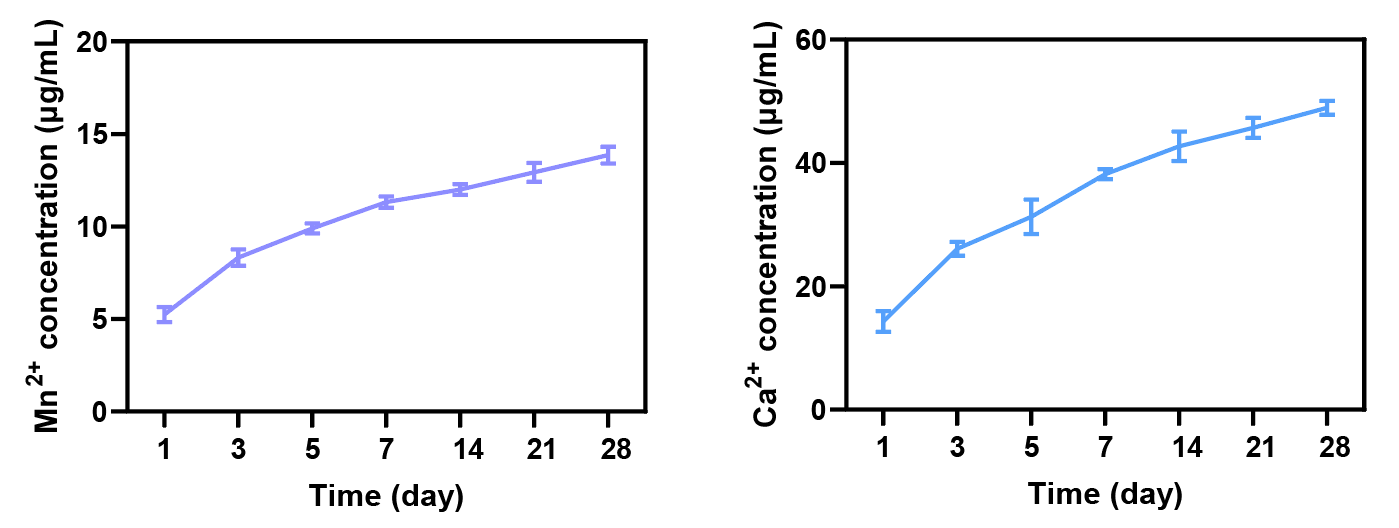


Figure S2 Ion release from MnCaP/HS


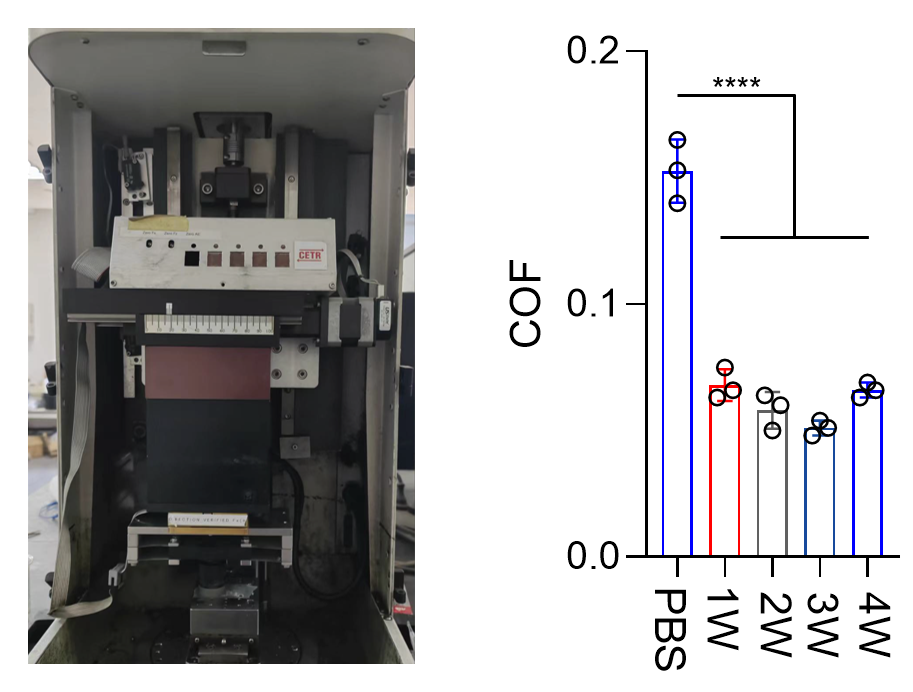


Figure S3 Friction coefficient of MnCaP/HS and HAMA (****p < 0.0001).


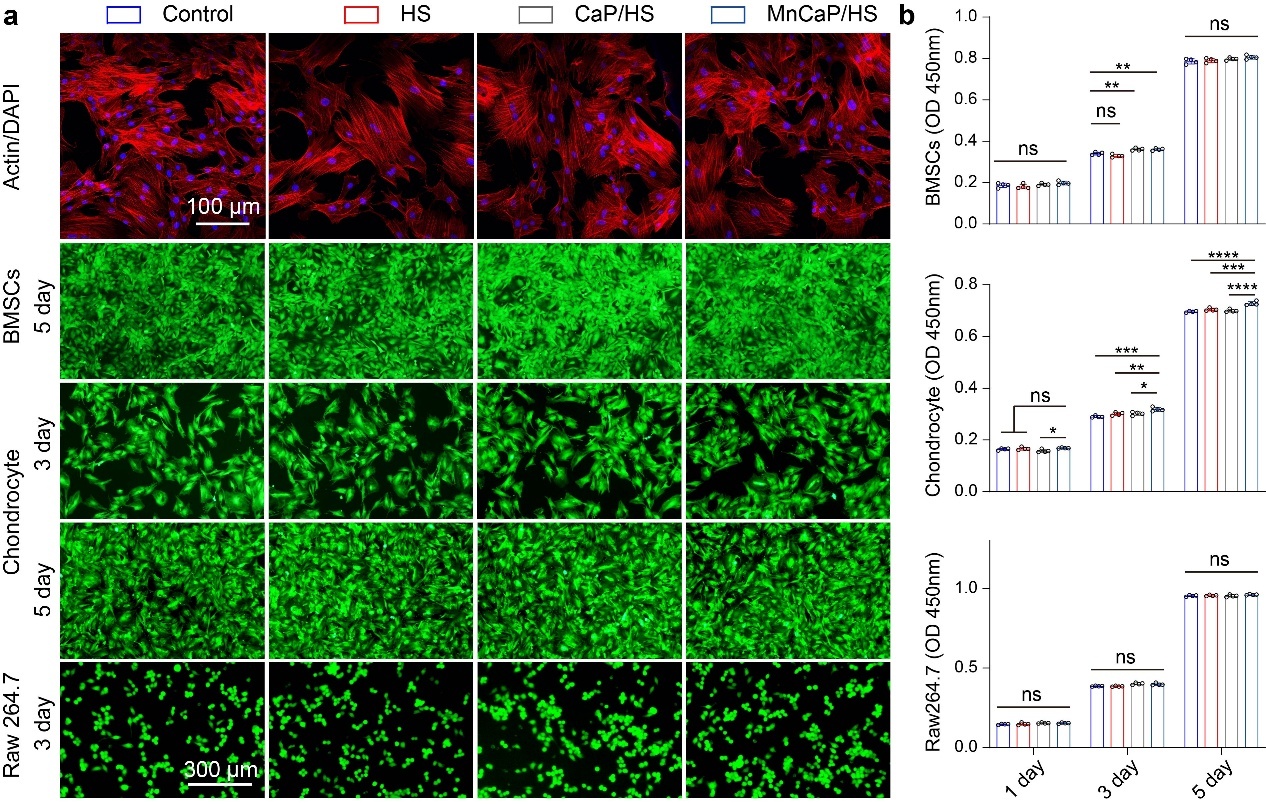


Figure S4. Biocompatibility of solid-liquid interface lubricating hydrogels. (a) Skeletal staining, live-dead staining and live-dead staining of BMSCs, chondrocytes and Raw264.7 cells after solid-liquid interface lubricating hydrogels treatment. (b) Cell viability of BMSCs, chondrocytes and Raw264.7 cells after solid-liquid interface lubricating hydrogels treatment at 1, 3 and 5 days. (ns, no significant difference, *p < 0.05, **p < 0.005, ***p < 0.001, ****p < 0.0001).


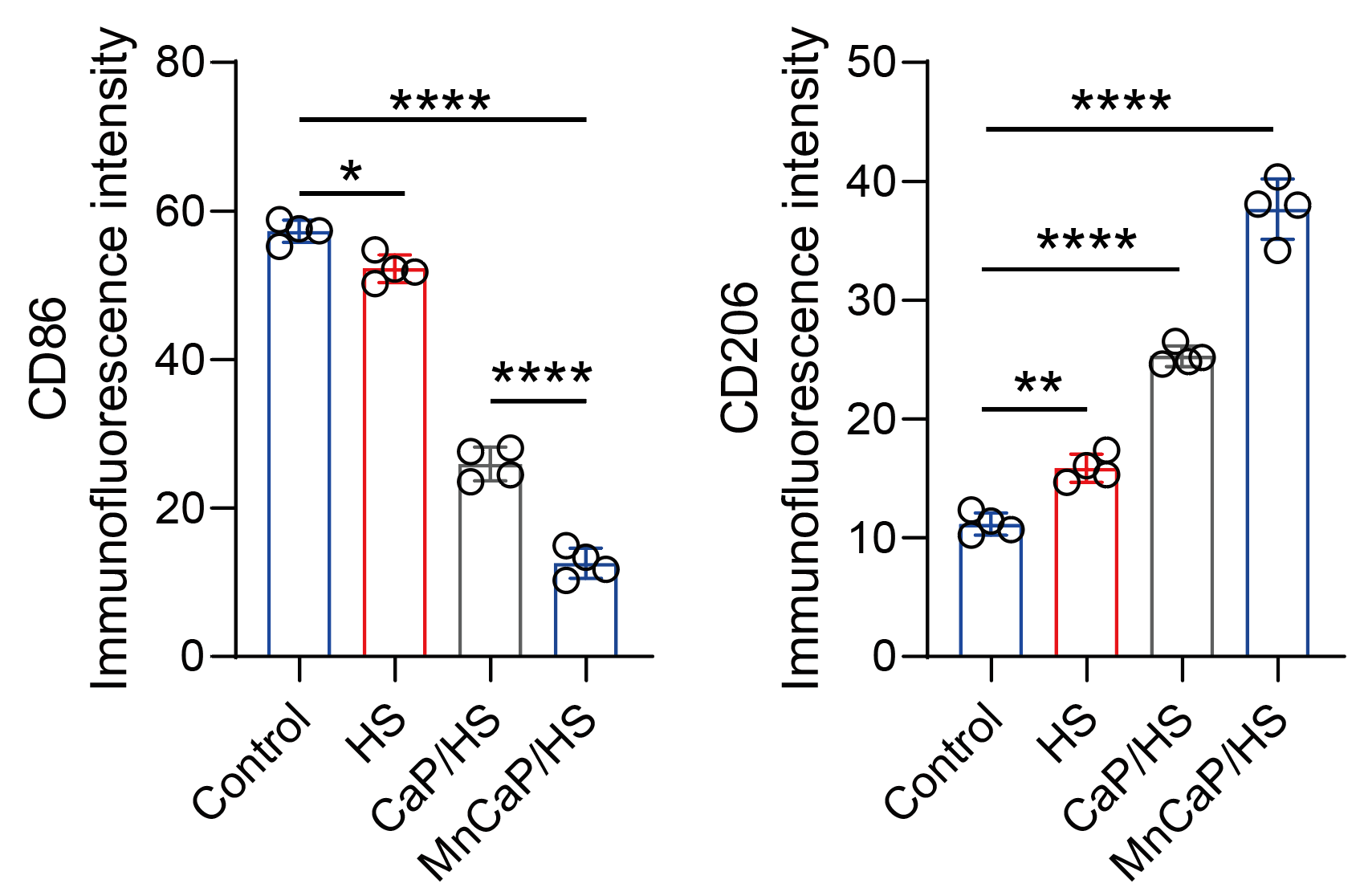


Figure S5 Fluorescence intensity of macrophages. (ns, no significant difference, *p < 0.05, **p < 0.005, ***p < 0.001, ****p < 0.0001).


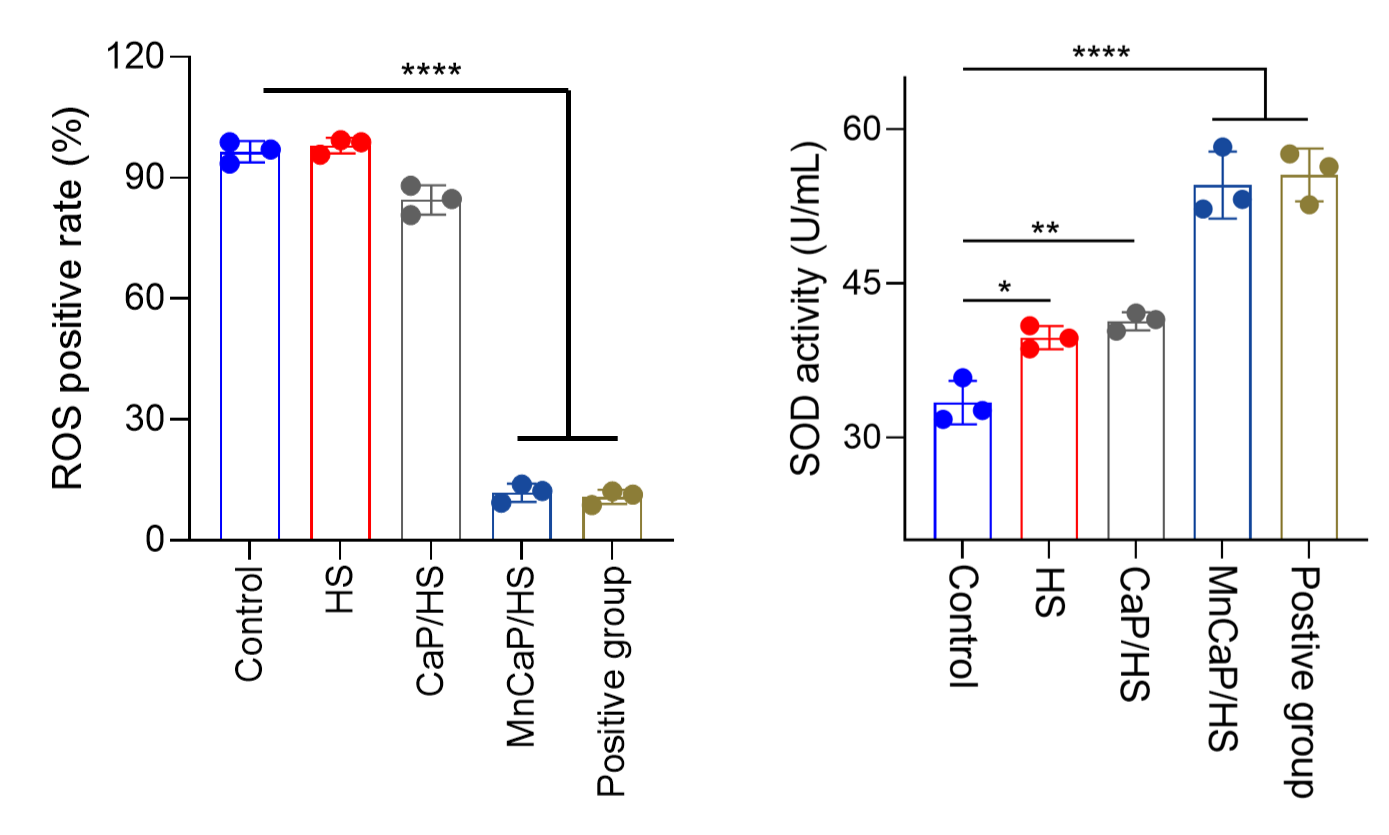


Figure S6 ROS positive rate and SOD activity. (ns, no significant difference, *p < 0.05, **p < 0.005, ***p < 0.001, ****p < 0.0001).


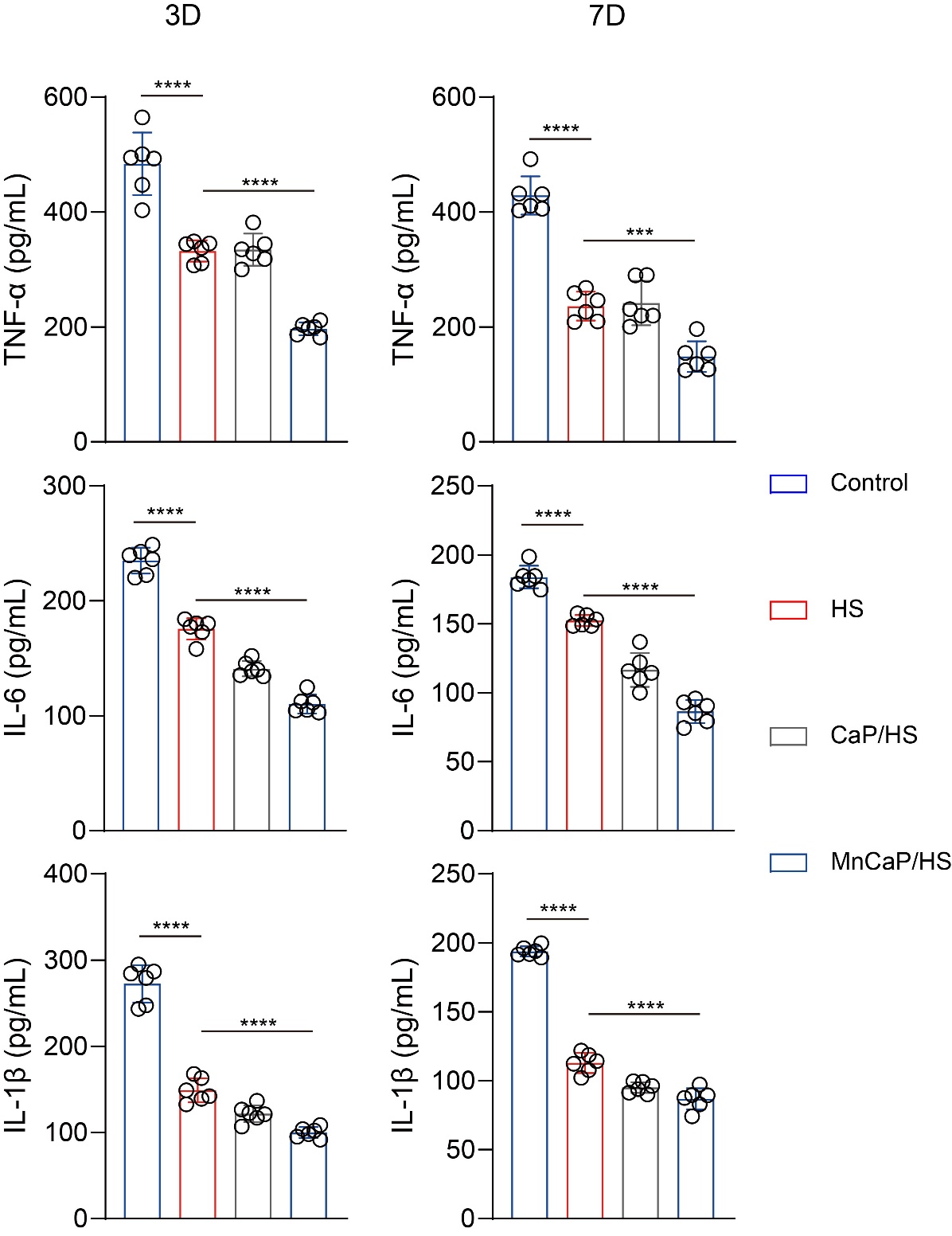


Figure S7 The levels of inflammatory factor at 3 and 7 Days. (ns, no significant difference, *p < 0.05, **p < 0.005, ***p < 0.001, ****p < 0.0001).


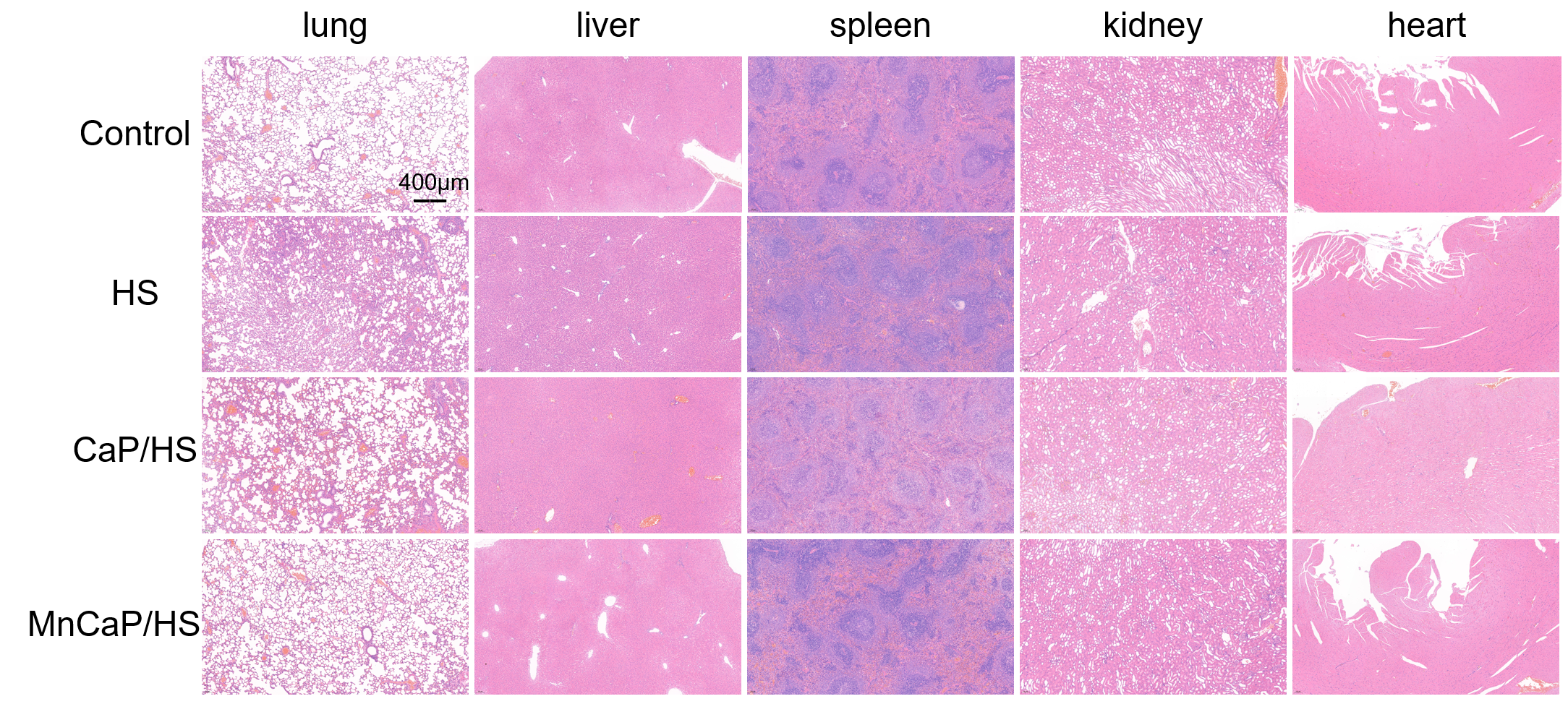


Figure S8 Heart, liver, spleen, lung, and kidney sections of rats in each group at 8 weeks


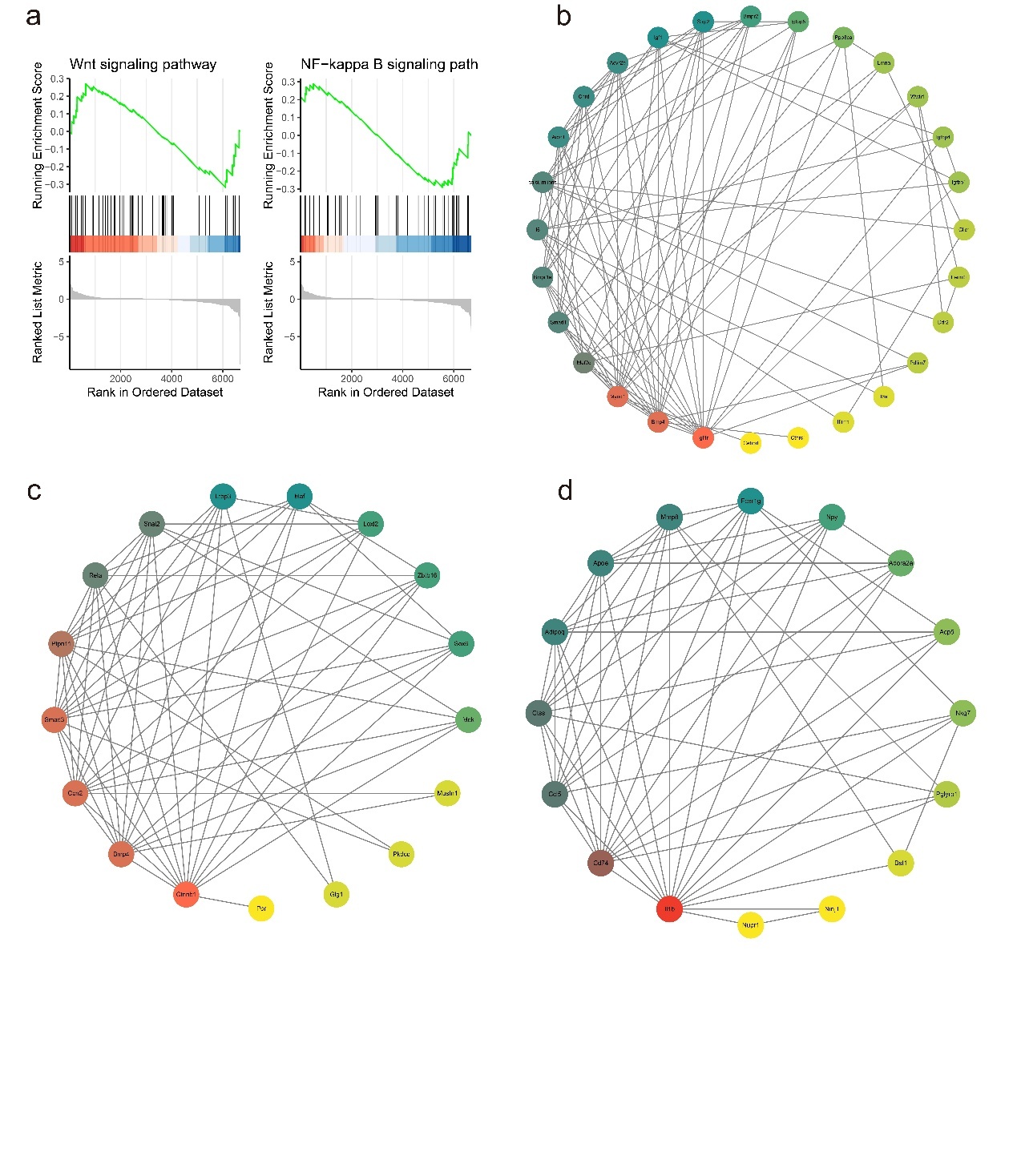


Figure S9 GSEA plots of the (a) Wnt and NFkB pathways and core gene interaction analysis of (b) osteogenesis, (c) chondrogenesis, and (d) inflammation

Table 1 Primer sequences

| Gen name | Primer sequence (5’ to 3’) |
| --- | --- |
| Col-Ⅰ | TGAGACAGGCGAACAAGGTGAC（Forward） |
|  | GGACCAGCAGGACCACTATCG（Reverse） |
| ALP | GGACCATTCCCACGTCTTCAC（Forward） |
|  | CCTTGTAGCCAGGCCCATTG（Reverse） |
| Runx2 | CCTCTGACTTCTGCCTCTGG（Forward） |
|  | GATGAAATGCCTGGGAACTG（Reverse） |
| OPN | GAGACCGTCTGAAACAGCGT（Forward） |
|  | AACCACTGCCAGTCTCATGG（Reverse） |
| OCN | ACAAGTCCCACACAGCAACTC（Forward） |
|  | CCAGGTCAGAGAGGCAGAAT（Reverse） |
| TGF-β1 | CTCCCGTGGCTTCTAGTGC（Forward） |
|  | GCCTTAGTTTGGACAGGATCTG（Reverse） |
| TNF-α | CACCACGCTCTTCTGTCTACTGAAC（Forward） |
|  | TGACGGCAGAGAGGAGGTTGAC（Reverse） |
| IL-6 | ATAGTCCTTCCTACCCCAATTTCC（Forward） |
|  | GATGAATTGGATGGTCTTGGTCC（Reverse） |
| IL-10 | GGACAACATACTGCTAACCGACTCC（Forward） |
|  | CTTCACCTGCTCCACTGCCTTG（Reverse） |
| Col-Ⅱ | CGTCTACCCCAATCCAGCAAA（Forward） |
|  | AGCAGGCGTAGGAAGGTCAT（Reverse） |
| SOX-9 | CACACTCCTCCTCCGGCATGA（Forward） |
|  | GCGGAAGTCGATAGGGGGCT（Reverse） |
| COMP | GAACGCTCTGTGGCATACA（Forward） |
|  | CAGGAACCAACGATAGGACTTC（Reverse） |
| ACAN | CCATCTCTACACGCTACACCC（Forward） |
|  | TTGTCTCCATAGCAGCCTTCC（Reverse） |
| Col-X | ACCCAAGGACTGGAATCTTTAC（Forward） |
|  | GCCATTCTTATACAGGCCTACC（Reverse） |
| INOS | CACCTTGGAGTTCACCCAGT （Forward） |
|  | ACCACTCGTACTTGGGATGC（Reverse） |
| Arg-1 | CTCCAAGCCAAAGTCCTTAGAG（Forward） |
|  | AGGAGCTGTCATTAGGGACATC（Reverse） |
